# Modelling charge profiles of electric vehicles based on charges data

**DOI:** 10.12688/openreseurope.14354.1

**Published:** 2021-12-23

**Authors:** Natascia Andrenacci, Federico Karagulian, Antonino Genovese

**Affiliations:** 1 Ente per le Nuove Tecnologie, l'Energia e l'Ambiente (ENEA), Rome, 00193, Italy

**Keywords:** Electric mobility, sustainable transport, policy, renewable energy, scenario analysis.

## Abstract

**Background: **The correct design of electric vehicle (EV) charging infrastructures is of fundamental importance to maximize the benefits for users and infrastructure managers. In addition, the analysis and management of recharges can help evaluate integration with auxiliary systems, such as renewable energy resources and storage systems. EV charging data analysis can highlight informative behaviours and patterns for charging infrastructure planning and management.

**Methods: **We present the analysis of two datasets about the recorded energy and duration required to charge Electric Vehicles (EV) in the cities of Barcelona (Spain) and Turku (Finland). In particular, we investigated hourly, daily and seasonal patterns in charge duration and energy delivered. Simulated scenarios for the power request at charging stations (CSs) were obtained using statistical parameters of the Barcelona dataset and non-parametric distributions of the arrivals. Monte Carlo simulations were used to test different scenarios of users’ influx at the CSs, and determine the optimal size of an integrated renewable energy system (RES).

**Results:** This study highlighted the difference between fast and slow charging users’ habits by analysing the occupancy at the charging stations. Aside from the charge duration, which was shorter for fast charges, distinct features emerged in the hourly distribution of the requests depending on whether slow or fast charges are considered. The distributions were different in the two analysed datasets. The investigation of CS power fluxes showed that results for the investment on a RES could substantially vary when considering synthetic input load profiles obtained with different approaches. The influence of incentives on the initial RES cost were investigated.

**Conclusions: **The novelty of this work lies in testing the impact of different simulated profiles as input in the economic criterion of the net present value (NPV) for determining the size of a photovoltaic (PV) system installed at a charging infrastructure.

## Plain language summary

The reduction of greenhouse gas emissions from road transport can be achieved with a transition to less polluting forms of vehicle power supply, such as electricity. To facilitate the large-scale adoption of electric mobility, charging an electric vehicle should be simple and reliable. To this end, an adequate charging infrastructure is required. However, we are faced with a typical chicken-and-egg problem: the number of users who switch to electric vehicles will be limited until a recharging infrastructure is available; on the other hand, investments in infrastructures require greater certainty about the adoption of electric vehicles. To facilitate planning and investment decisions, it is important to evaluate the demand, through both quantitative and behavioural aspects. In the early stage of low uptake of electric vehicles, this assessment can be more difficult. In this work, we present an analysis of some charging data for two European cities: Barcelona (Spain) and Turku (Finland). We analysed the convenience of some types of investments for charging infrastructures based on the hourly charging profiles obtained. The results show that the choice of profiles to be adopted in the analysis greatly influences the results, especially when the available data are limited.

## Introduction

Electric vehicles (EVs) represent the main answer for the abatement of greenhouse gas emissions in the transport sector ^1^. The introduction of financial incentives from governments led to an increase in the number of EVs sold in many countries ^2, 3^. Therefore, it is necessary to strengthen the network of electric charging stations (CSs) with adequate infrastructures to satisfy the charge demand. At this stage, careful planning of charging stations is crucial to meet user demand, ensure an economic return for charging point operators, and to guarantee the operability of the electrical grid. For this purpose, accurate forecasts of the energy demand at CSs are fundamental for optimal planning and operation. Clearly formulating the problems to address regarding the specifications of the infrastructure of interest is required ^4^. For instance, the charge demand from commercial fleets is strictly related to the type of transport activity ^5, 6^. For this work, we only considered the charge demand related to the private transport activity.

Forecasting energy demand through charging operations can be based on users’ habit and on historical traffic data related to EVs. Statistical analysis allows inferring some probabilistic trends for the expected charge demand through suitable probability distribution functions ^7^. Charging habits include various information, such as the usual charging start times and duration, the amount of charge required, the state of charge (SOC) of the battery and the type of car. Based on time series of charging data, forecasting methods often use stochastic or machine learning approaches to predict future charge demand ^8^. In addition, numerical techniques like Monte Carlo (MC) simulations have been used to create synthetic charging profiles ^9^. On the other hand, parking data has also been used to determine the charge demand through the assumption that the charge probability increases with parking time and decreases if charges were made earlier in the same day ^10^. Within this context, the potential daily load of an EV was evaluated through traffic/parking simulations using agent-based models ^11^. Modelling the arrival of EVs at the charging stations, together with energy request, is usually simulated using a stochastic model based on the so-called Markovian Queueing model as birth and death process (M/M/c/k) ^12^. In this approach, the arrival rate is modelled as an inhomogeneous Poisson process with one peak in the morning and one in the evening, while the charging rate is modelled as a homogeneous Poisson process. Similar work simulated arrivals of EVs at the charging station using the General Markovian model M/G/k queue ^13^. In this approach, a random Poisson process models arrivals, while the charge duration is fixed and the energy demand for each EV is approximated by a Gaussian distribution.

Other approaches forecasting the charge demand used real traffic flow data. Previous work showed the load profile was obtained using GPS data from private vehicles circulating in the urban area and assuming a transition of a fixed percentage of users from fuel-powered to electric cars ^14^. The energy request was estimated from the evaluation of the consumption and instantaneous speed of a medium-size EV required for each trip. In another work ^15^, real-world traffic data were combined with weather data to determine travel patterns, which may affect the EV charging demand forecasting. In that case, EV SOC and “start” charging time followed a Gaussian distribution. Alternative data-driven approaches, estimating EV charging demand, used traffic flow data and travel patterns extracted from OpenStreetMap and from a battery capacity prediction model ^16^; alternatively, they used online ride-hailing trip data to forecast charging demand regardless of whether data was referring to electric or conventional vehicles ^17^.

As shown above, forecasting the energy demand at the charging station is usually accomplished using time-series of historical data. Recent work ^18^ analysed and compared different forecasting models, such as Auto-Regressive-Moving-Average, autoregressive integrated moving average, artificial neural networks, and long short-term memory modelling. This study highlighted the uncertainties of the forecasting process related to the quality and amount of accessible data. This issue can be partially overcome using a short-term load forecasting model based on Support Vector Machines ^19^. Another issue arises from the time distribution and magnitude of energy demand that often comes from multiple sources. Therefore, data formats may be heterogeneous and the availability dependent on different recording rates. This issue can be overcome by using a distributed and dynamic computing architecture consisting of a series of autonomous phases, in which data from different sources are combined and made available by a regulating authority ^20^.

## Methods

For this work, we analysed EV charging datasets provided by two cities participating in the User-Chi project ^21^. This project focuses on the design of electric charging networks satisfying user needs with the aim of developing marketable, innovative and highly convenient charging systems. In the following, we present the charging events datasets and statistical analyses.

Datasets relating to the registration of charging events were collated during the year 2019 by the Municipal Area of Barcelona (“Area Metropolitana de Barcelona”, AMB) in Spain, and the city of Turku in Finland. The two datasets were heterogeneous in structure and information, and had the following structures:

•       The AMB data refers to public charging points, managed by the municipality, and is divided in two subsets:
  a) The first subset contains information about the charging events, such as charging point (CP) ID, connector type, charge start time; charge stop time, charge duration (minutes), energy delivered (kWh), vehicle manufacturer (optional) and, model (optional);
  b) The second subset contains information about the CP such as: location (address), longitude, latitude, typology of connectors and charging mode at the charging point (i.e.: Schuko 3kW 16A mode 1, Mennekes 7 kW 16A mode 3, Mennekes 43 kW 63A mode 3, CHAdeMO 55kW 125A mode 4), and charging point makers.
  In this data, “charging point” refers to a CS that contains more than one plug at which an EV can be charged. In the following, we will refer to “slow” chargers for 3 kW and 7 kW charging points and to “fast” chargers for 43 kW and 55 kW charging points.

•       The Turku dataset contains information about charging events at public charging points and contain the following information: station ID, station name, charge start time, charge stop time, charge duration, energy delivered (Wh), plug type (alternative current [AC] or direct current [DC]), and cumulative energy delivered (Wh). AC refers to 22 kW chargers operating with alternating current, while DC refers to 50 kW chargers operating with direct current.

The definition of “slow” and “fast” charges is not straightforward. Indeed, if we consider charging time, “slow” charges take one or more hours to complete, while “fast” charges are able to refill up to 80% of the battery capacity in about a half-hour ^22^. Clearly, this classification of “slow” or “fast” depends on both the charging power and the battery size, and can be replaced by other definitions. For example, recent work ^23^ considered as “fast” all chargers with a power level equal or greater than 36 kW, regardless of whether it was delivered with alternating or direct current.

The classification of the charger types can refer to the maximum power level and to the connectors used. Chargers can be classified according to three different levels, as reported in Table 1.

**Table 1.** Main characteristics of different charger levels (‎ 24– 26). AC: alternative current; DC: direct current.

Power level	Typical use	Voltage	Typical power	Charging time
Level 1	Home, work	120 AC	2 kW	4–11 h
Level 2	Home, work, public	208–240 AC	7 kW 20 kW	1–6 h
Level 3	DC fast – ultrafast	480–900 DC	100 kW	< 30 min

Level 1 is typically implemented at residential sites and it can be accomplished without specific equipment. The connector adopted in Level 1 is the J1772. However, this type of charging is not present in Europe ^25^. On the other hand, Europe adopted the Mennekes IEC 62196 Type 2 connector, which meets the specification for Level 2 ^26^. Finally, for Level 3, the connector depends on car manufacturers: Japan the Charging de Move (CHAdeMO) is standard; other manufacturers use the Combined Charging System (CCS) or “Combo” plug; in China, the Guobiao recommended standard (known worldwide as GB/T) is used, while the brand Tesla uses its proprietary plug. In the following, we will refer to a single charge plug as a charging point (CP).

## Results and discussion

### AMB dataset (Barcelona)

The AMB dataset contains the charging registrations at each CP. This is combined with another dataset containing information about the CS. The CSs are identified by an ID and can be divided into three types:

1. 11 CSs with two Schuko 3kW CPs;
2. 10 CSs with one Mennekes 43 kW CP, one CHAdeMO 55kW CP and one Combo CCS 55 kW CP
3. one CS with two Mennekes 7 kW CPs.

In the following, charges of EVs at the Schuko 3kW and Mennekes 7 kW CPs are referred to as “slow”, while the other ones as “fast”.

We also defined a single charging infrastructure (CI) as the set of CS identified by the same address and geographic coordinates. Consequently, we obtained:

1. 10 CIs that included two Schuko 3kW CPs, one Mennekes 43 kW CP, one CHAdeMO 55kW CP, and one COMBO CCS 55 kW CP.
2. One CI that included two Mennekes 7 kW CPs and two Schuko 3kW CPs.

The original dataset consisted of about 38,000 records classified as charging registrations. Records with zero energy exchange (around 4.7% of the dataset) were removed from the dataset. In addition, records with average charging power, defined as the ratio between energy delivered and charge duration, greater than the maximum nominal power of the CP, were also removed. Table 2 reports statistical figures on the usage of the CPs based on their typology, such as the daily and yearly average number of charges as well as the maximum and minimum number of daily charges.

**Table 2.** Typical usage of a charge station based on its typology. CP: charging point.

CPs typology	N charge/year	Maximum charge/day	Minimum charge/day	N charge/day
Slow	286.7	1.69	0.15	0.79
Fast	3302.9	14.03	3.78	9.05

**Figure 1.**
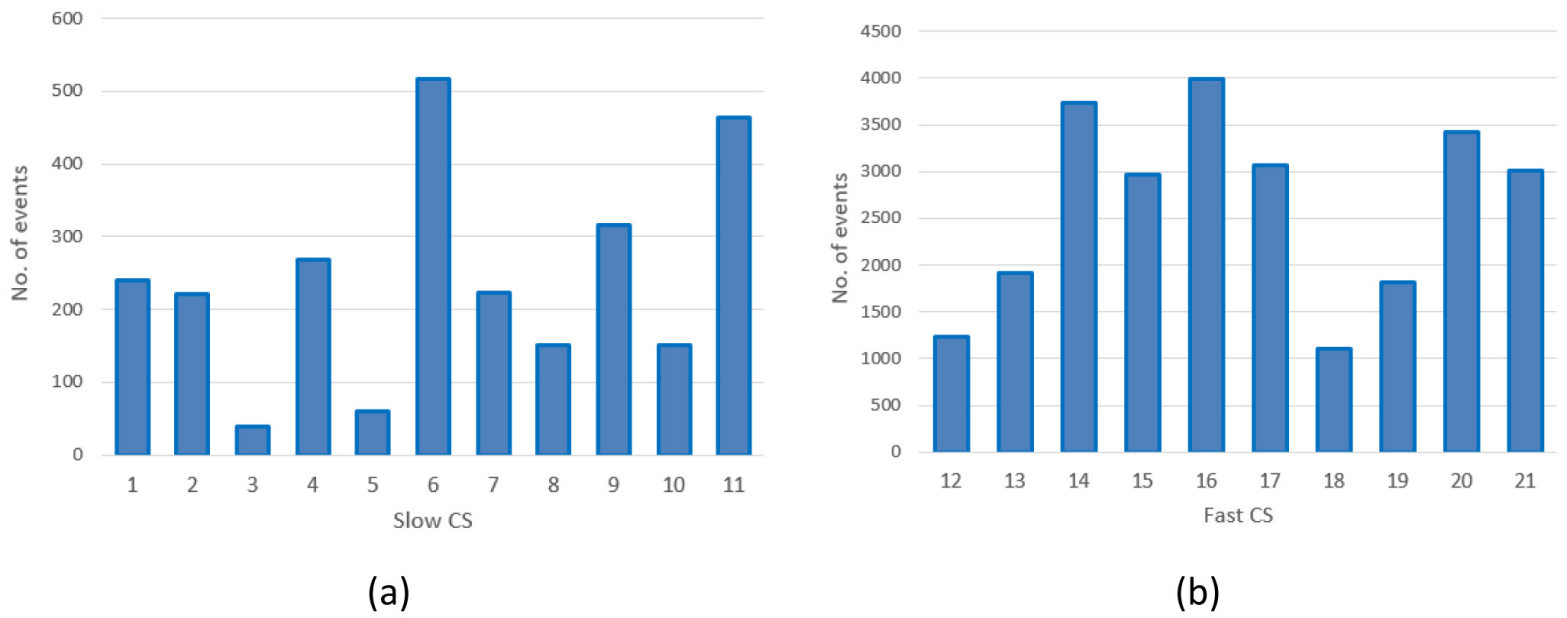
Number of charges in 2019 at slow charging stations (CS) ( **a**) and fast CSs ( **b**) for the Area Metropolitana de Barcelona (AMB) dataset.

The distribution of the charges among CSs is illustrated in Figure 1. With the exception of the CS labelled as 1, corresponding to CI number ‎ 2, only equipped with slow chargers, all the other CIs were composed of CSs labelled *x* and *x+10* for the slow and fast CSs, respectively (for example, slow CS number 2 and fast CS number 12 correspond to the same CI. Table 3 reports the number of stations present at each location, classified as slow or fast CS, with relative usage.

**Table 3.** Usage of charge stations at each location.

CI name	Slow CS Id	No. Of charges (year)	Fast CS Id	No. Of charges (year)	Total no. Of charges (year)
Sant Andreu da la Barca: Pg. Rafael de Casanova FGC	1	280			280
Badalona: C. Anna Tugas - Pg. Olof Palmer	2	269	12	1582	1851
Barberà del Vallés: C. Arquímedes, 8	3	55	13	2399	2454
Cornellà de Llobregat: Carrer de Baltasar Oriol i Mercer	4	315	14	4679	4994
El Prat de Llobregat: Pl. Volateria (Mas Blau)	5	72	15	3704	3776
Gavà: C. del Progres, 54	6	616	16	5121	5737
L'Hospitalet de Ll.: C. Salvador Espriu - Gran Via de les Corts Catalanes	7	257	17	3824	4081
Montcada i Reixac: C. Tarragona - C. Pla de Matabous	8	171	18	1378	1549
Pallejà: Rda. Santa Eulalia - C. Joan Maragall	9	397	19	2389	2786
Sant Cugat del Vallès: Av. Via Augusta, 3	10	201	20	4305	4506
Sant Joan Despí: C. TV3 - C. Jacint Verdaguer	11	521	21	3648	4169

As shown in Figure 1, the average daily demand for slow CSs was lower than one user per day, with the exception of three stations (number 6, 9 and 11) that exceeded 365 users per year. Instead, as shown in Figure 1(b), the demand for fast CSs was always greater than three users/day, and the stations number 14, 16 and 20 were the Busiest.

The average charging duration was estimated to be of about 42 minutes with a standard deviation of about 90 minutes. On the other hand, the average energy delivered was about 10 kWh with a standard deviation of nearly 7.8 kWh ( Table 3). When selecting only slow chargers (3kW or 7kW), we obtained an average charging duration of about 182 minutes with a standard deviation of about 263 minutes, while the average energy delivered was about 4 kWh with a standard deviation of about 5 kWh ( Table 3). The large value obtained for the standard deviation was due to the presence of several charging events with a duration longer than a day.

If only fast (43 kW AC and 50–55 kW DC) chargers were selected, the average charge duration was about 29 minutes with a standard deviation of about 18 minutes and a median value of 26 minutes. On the other hand, the average energy delivered was about 10.7 kWh with a standard deviation of 7.7 kWh ( Table 4).

**Table 4.** Statistical parameters for Area Metropolitana de Barcelona (AMB) datasets. Stdev: Standard deviation.

Dataset	Number of records/charges	Mean charging duration (min)	Stdev duration (min)	Mean energy (kWh)	Stdev energy (kWh)
Entire	36192	41.99	90.37	10.09	7.76
Slow	3163	181.56	262.96	4.11	4.91
Fast	33029	28.67	17.57	10.66	7.74

**Figure 2.**
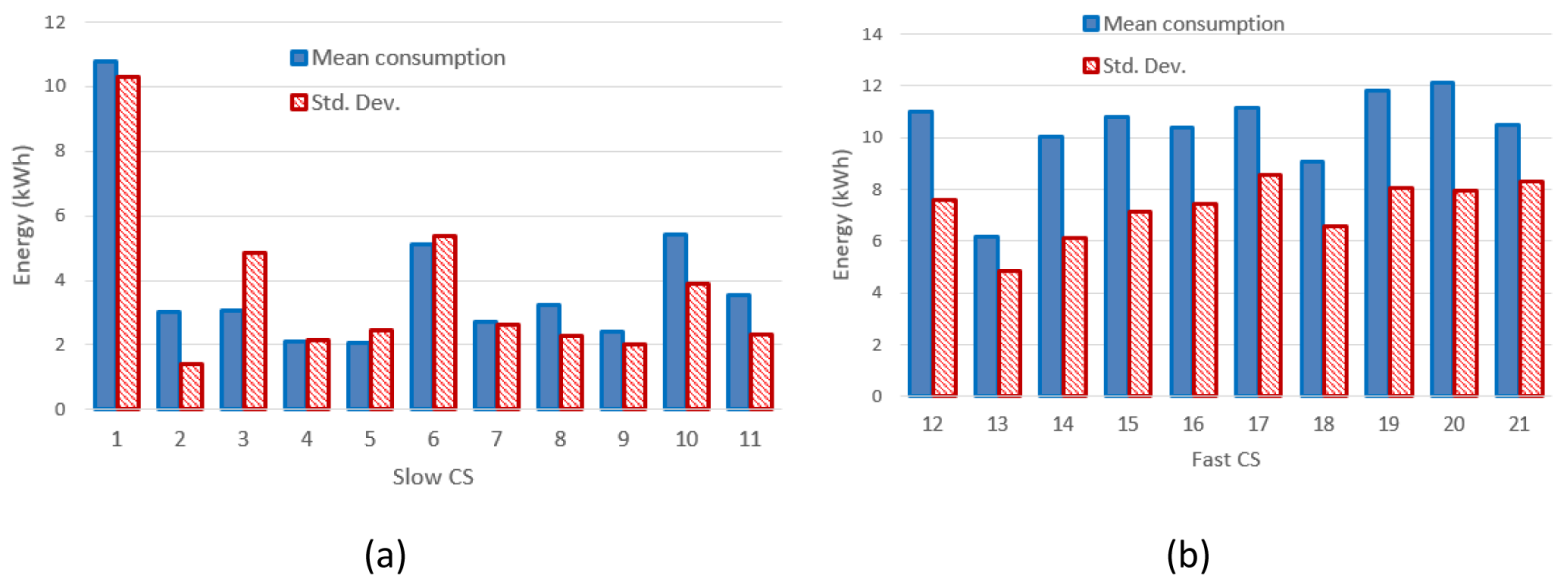
Mean value and standard deviation (Std. Dev.) of the energy delivered during the charge for slow charging stations (3 and 7 kW) ( **a**) and fast charges ( **b**).

The mean value and the standard deviation for the energy at each CP are shown in Figure 2(a) for slow CPs and Figure 2(b) for fast CPs. 

**Figure 3.**
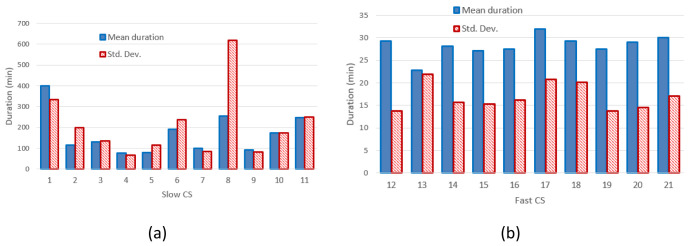
Mean charging duration and standard deviation (Std. Dev.) for slow ( **a**) and fast ( **b**) charging stations (CSs).

Figure 3(a) and Figure 3(b) report the mean value and the standard deviation of charge durations for slow and fast CPs, respectively.

Figure 2 clearly shows that the distribution of the mean energy delivered during the charging operation was more homogeneous for fast chargers when compared to slow chargers. The same behaviour was observed for the distribution of the mean charging duration ( Figure 3). This suggests the fast charge “behaviour” could be predicted with a higher degree of confidence compared to the slow one.

**Figure 4.**
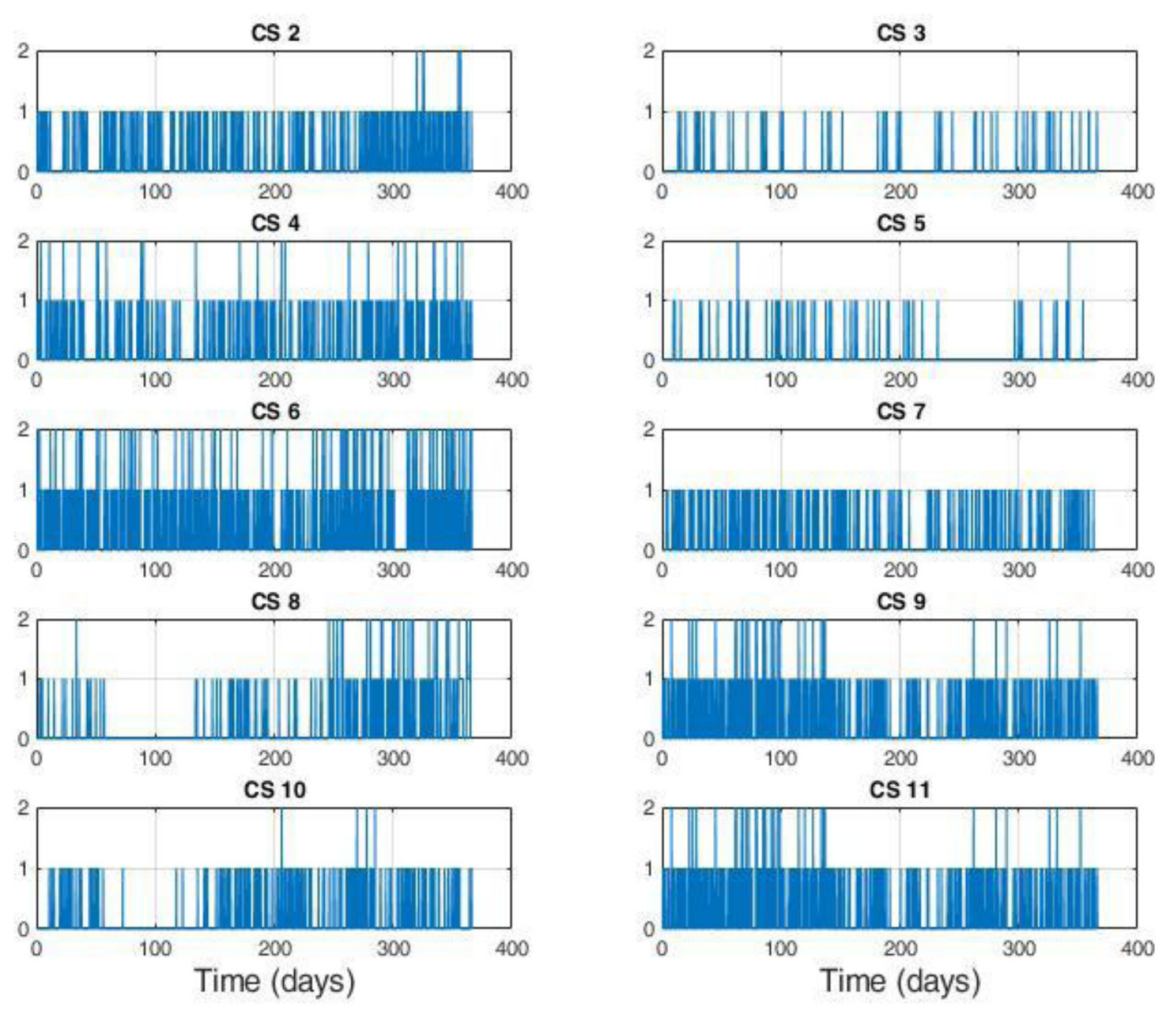
Number of users at slow charge stations (CSs). Each station is equipped with two charging points (CPs).

Analysing the number of users present at the CPs during each day of the year 2019, we found the four available CPs (two 7 kW CPs and two 3kW CPs, station ‎3) were never simultaneously occupied, and the maximum occupation rate was three at a time. Similarly, we found that fast charging stations were never fully deployed, as the occupancy was never greater than one, although there were three CPs in each station. Finally, as shown in Figure 4, the occupancy at slow charging stations sometimes saturated the two available CPs. However, this could be due to a parking duration longer than the charging time.

**Figure 5.**
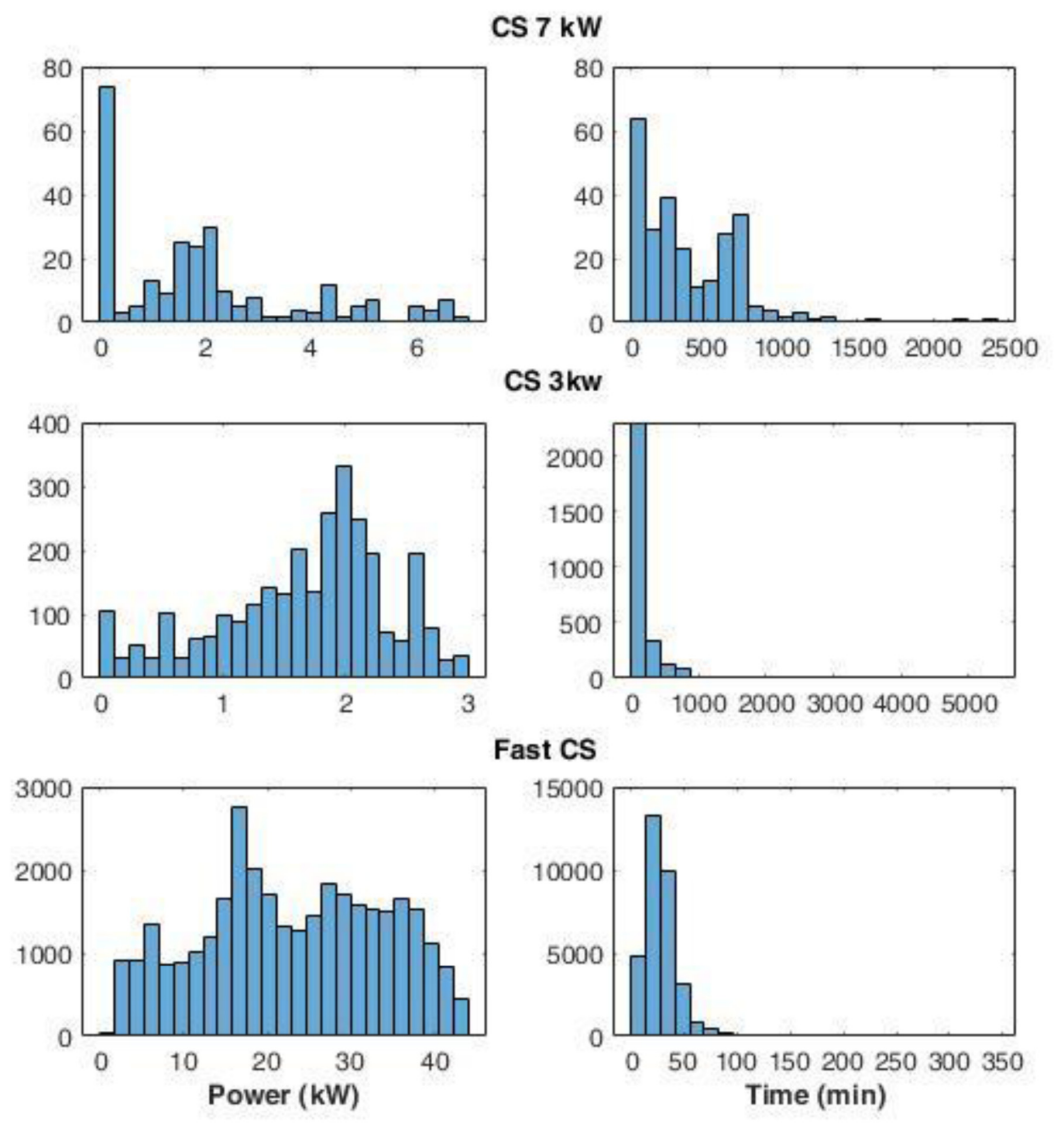
Distribution of the average power delivered during charging (left) and charge duration (right) at 7 kW station, 3 kW stations, and fast stations.

The average power delivered during the charges was defined as the ratio between the energy delivered and the charge duration. For the CS at 7 kW (slow charging), we found a large number of events at very low average power (below 0.2 kW) ( Figure 5). A possible explanation could be that EVs remained connected to the charger even after their charge ended. On the other hand, analysing the distribution of the fast and slow (3 kW) CSs, we observed maximum peaks at 17 kW and 2 kW, respectively.

Interestingly, some events using slow chargers were able to deliver up to 3 kW ( Figure 5) that corresponded to the maximum available power at that station. On the other hand, at the fast-CSs, no event delivered the maximum available power of 55kW. This is due to the maximum power only being delivered when the battery could accept it, which only happens when the battery is large enough and the SOC is sufficiently low. The distribution of charge duration at 7 kW CS showed the most pronounced peak between 0 and 100 minutes, while two other peaks were found at 200 and 700 minutes ( Figure 5). Overall, 96% of the charge duration lasted less than 800 minutes. Fast and 3 kW CS charge duration distributions presented a Poisson-like form, with long tails: for 3 kW CSs, 83% of charge durations were below 250 minutes, and the percentage rose to 98% if we considered the interval until 800 minutes. Finally, for fast charges, 66% of the durations were within the 15–40 minutes.

**Figure 6.**
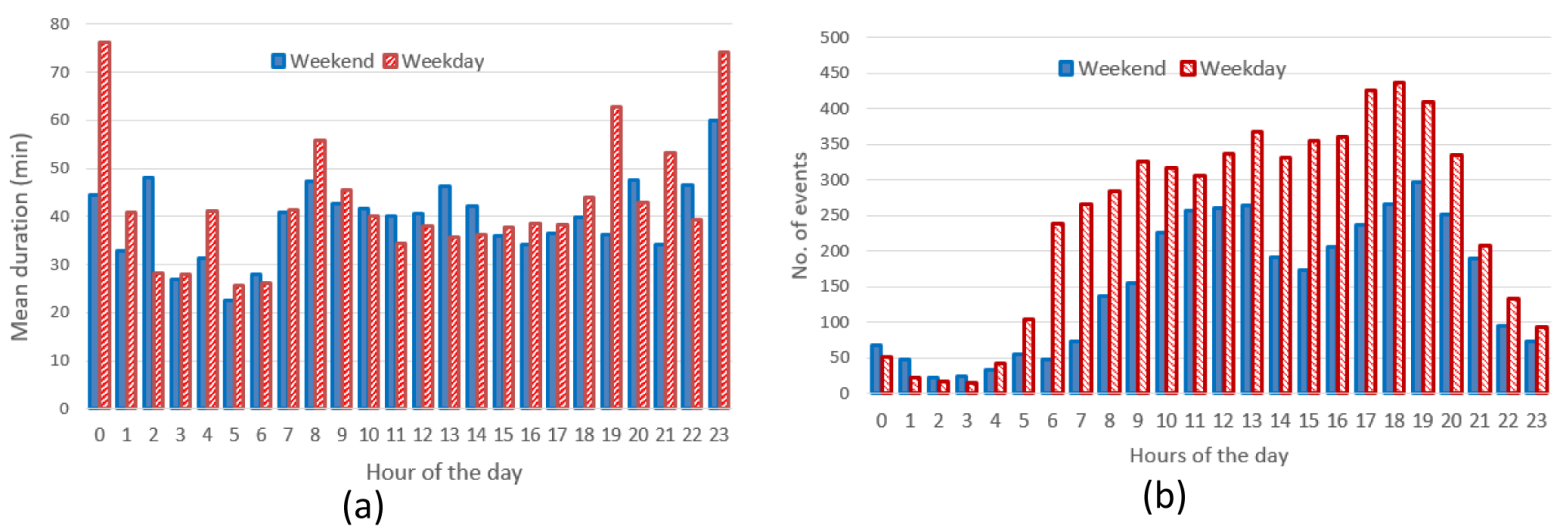
Average duration ( **a**) and number of charges ( **b**) distributions for slow and fast chargers together.

***Daily and seasonal effects on charges distribution.*** The hourly distribution of the mean duration of the charges at their starting time for both slow and fast chargers, shows that during night-time, charges tended to be longer compared to early morning during weekdays ( Figure 6 (a)), while for weekend charges this tendency was less pronounced. On the other hand, the hourly distribution of the number of the charging events at their starting time, for weekdays (Monday to Friday) and weekends (Saturday and Sunday), showed that charges were mainly concentrated between 7 am and 10 pm, with a peak around 6 pm ( Figure 6 (b)).

**Figure 7.**
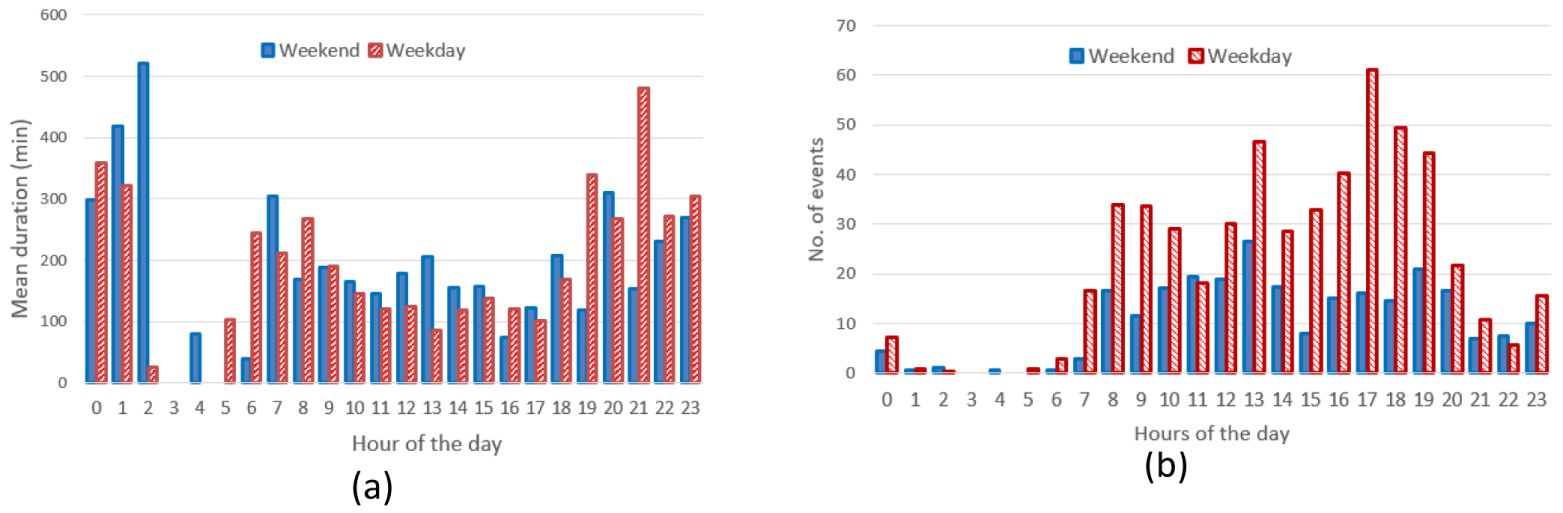
Average duration ( **a**) and number of charges ( **b**) distributions for slow chargers as a function of working day or weekend day hours.

Through analysing the distribution of the number of charging events, we observed a reduction of the number of charges during weekends compared to weekdays, with a little shift toward later hours (7 am–9 pm during weekdays, 11 am–10 pm during weekends). On the other hand, while analysing previous parameters for slow chargers during working days and weekend days ( Figure 7), the mean charge duration and number of charges did not considerably change during a typical working or weekend day. The distribution of the starting hour showed some shifts in the peaks and a reduction of the number of charges during the weekend.

**Figure 8.**
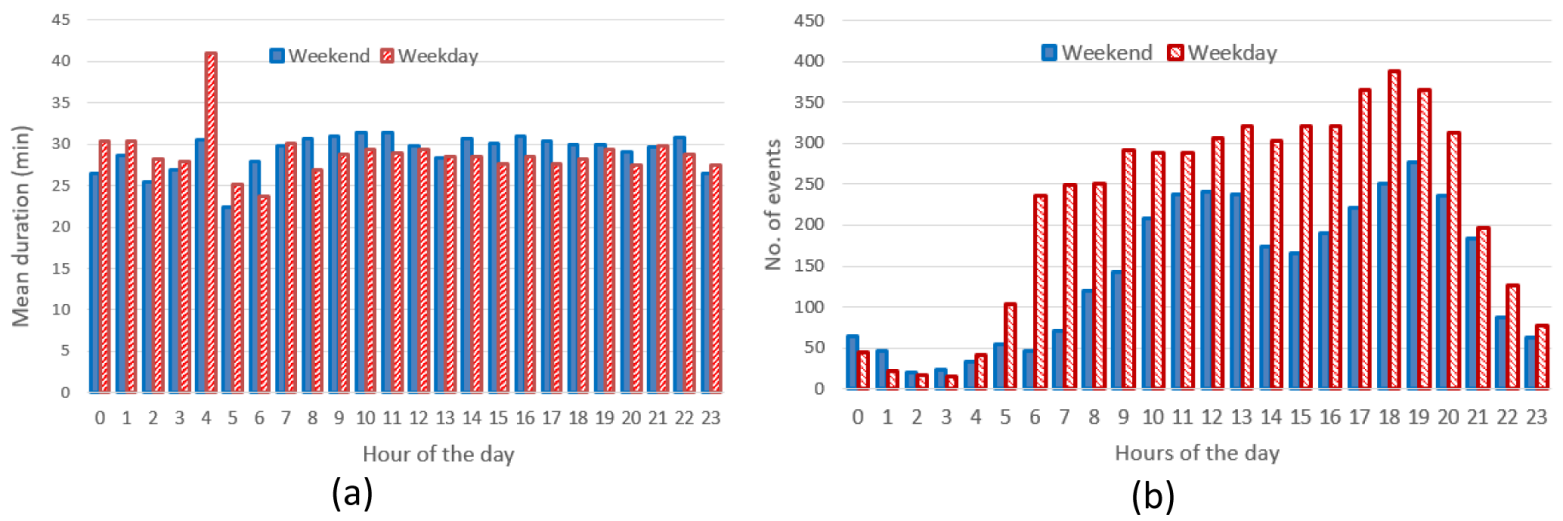
Average duration ( **a**) and number of charges ( **b**) distributions for fast chargers as a function of working and weekend day hours.

The analysis for fast chargers showed that the mean charge duration was quite homogeneous for different hours and days with a slight increase during the night-time of working days (12 pm – 5am) ( Figure 8). Instead, the number of fast charges was larger during an average working day rather than during an average weekend day, with charges mostly occurring during daytime (from 7 am to 9 pm for working days and from 11 am to 10 pm during weekend days). 

The distributions of start time for slow and fast charges were similar with a peak at late afternoon for workdays and two peaks, around noon and in the late afternoon, during the weekends. As shown in Table 5, the average daily number of charges during weekends was estimated to be about 64% and 47% of the average number of charges during working days, for fast and slow chargers respectively.

**Table 5.** Average parameters for weekend and working days.

Dataset	N of events/day/CS Monday-Friday	N of events/ day/CS Saturday and Sunday	Mean duration Monday-Friday (min)	Mean duration Saturday and Sunday (min)	Mean energy Monday- Friday (kWh)	Mean energy Saturday and Sunday (kWh)
Slow	0.94	0.44	228.01	176.92	4.18	3.97
Fast	10.1	6.5	28.42	29.87	10.59	11.86

**Figure 9.**
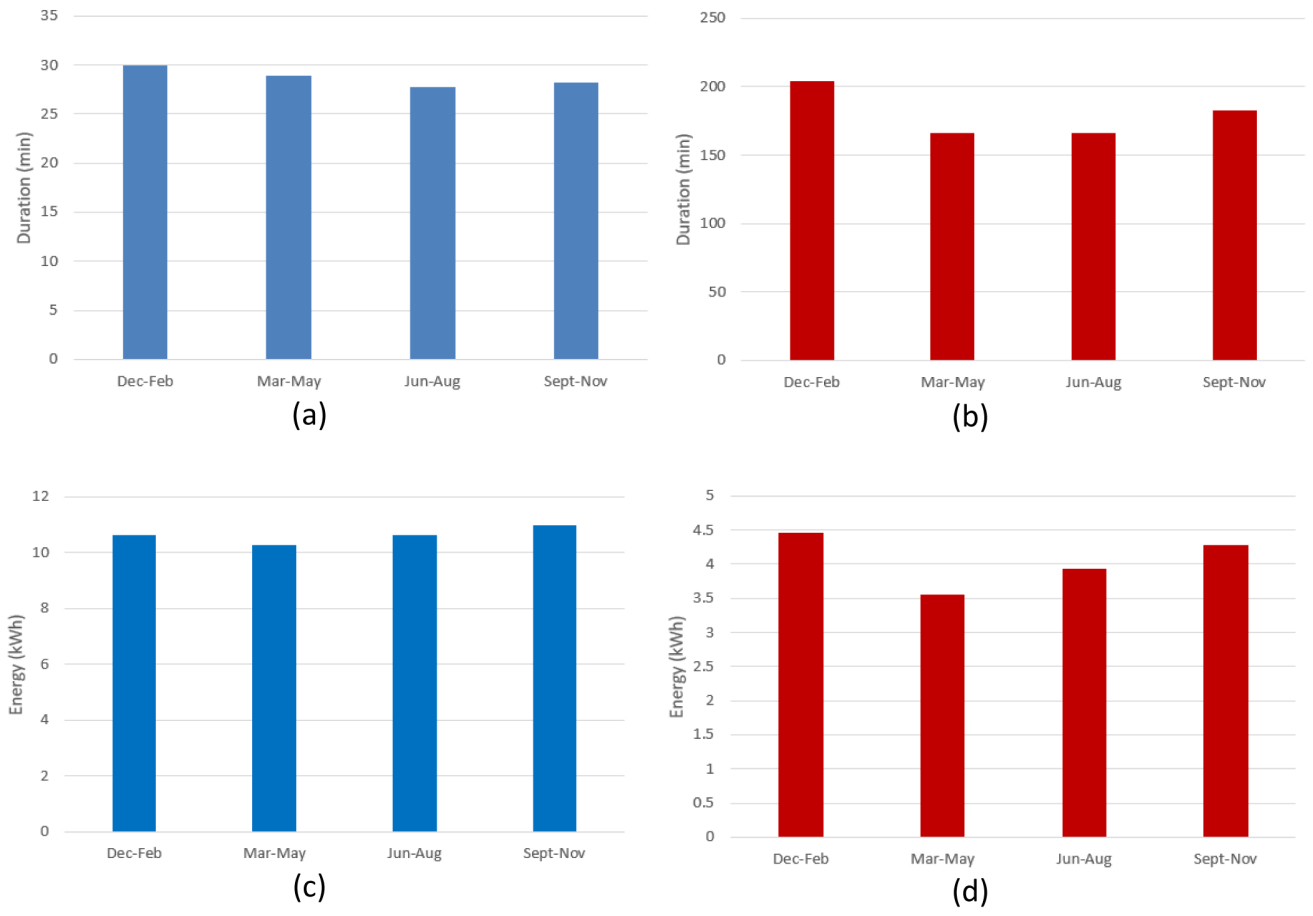
Seasonal variation of average charging parameters: ( **a**) fast charge duration; ( **b**) slow charge duration; ( **c**) fast charge energy consumption; ( **d**) slow charge energy consumption.

Figure 9 shows the seasonal mean duration and mean energy consumption of fast and slow chargers. The difference between the longest duration (in winter [December-February]) and the shortest duration (in summer [June-August]) was estimated to be about 7.5% and 18.5% for fast and slow chargers, respectively ( Figure 9 (a) and Figure 9 (b)). On the other hand, the difference between the highest (fall [September-November]) and lowest (spring [March-May]) energy delivered was estimated to be about 6.6% and 20% for fast and slow chargers, respectively ( Figure 9 (c) and Figure 9 (d)).

The shorter duration observed for fast chargers during the summer, compared to the longer one observed during the winter, could be related to fast charges requiring a longer time to be completed at low temperatures ^27^. On the other hand, the different seasonal trend observed between slow and fast charges might be related to the highly random behaviour in the slow charge usage.

***Correlation among EV battery size and charge parameters*.** Some charging registrations in the AMB dataset included information about the EV model plugged into the charging station. Further information was gathered from factory datasheets or technical journals.

We combined data extracted from the AMB dataset for registered EV models with battery information retrieved from factory datasheets or technical journals ^28^. Battery data were usually available for the latest models. Some EV models can have on-board batteries of different size. In that case, we chose to include only the battery of greatest size. The smallest battery size was 3.1 kWh for the Volta BNC, while the greatest size was 100 kWh for Tesla X and S. The collected data are reported in Table 6.

**Table 6.** Information on battery for the EV models. This table was reproduced from the AMB dataset and kept uncorrected, and therefore includes missing or duplicate misspelt manufacturer names.

ID	Manufacturer	Model	Battery (kWh)
1	Mitsubishi	OUTLANDER	13.8
2	Mitsubishi	OUTLANDER PHEV	13.8
3	Mitsubishi	I MIEV	16
4	Mitsubishi	I-MIEV	16
5	Nissan	E-NV200	22
6	Nissan	ZE0 /A/A02	24
7	Nissan	LEAF	51
8	Opel	AMPERA ELÈCTRIC	60
9	Peugeot	ION	16
10	Peugeot	PARTNER FG L1	20.5
11	Renault	TWIZY	6.1
12	Renault	TWIZY 45	6.1
13	Renault	FLUENCE	22
14	Renault	FLUENCE Z.E.	22
15	Renault	ZOE 240	22
16	Renault	KANGOO	31
17	Renault	KANGOO EXPRESS ZE	31
18	Renault	KANGOO ZE	31
19	Renault	ZOE	47
20	Smart	BRAUS ELECTRIC	16.7
21	Smart	ELECTRIC DRIVE	16.7
22	Smart	FORFOUR	16.7
23	Smart	FORTWO ELECTRIC	16.7
24	Tesla	MODEL 3	75
25	Tesla	S 90D	90
26	Tesla	MODEL X	100
27	Tesla	S	100
28	Tesla	S 4X4	100
29	toyota	PRIUS PLUG-IN	4.4
30	Vectrix	VX1	8
31	VolkWagen	E-UP!	25.5
32	VolkWagen	E-GOLF	55.7
33	VolkWagen	GOLF	55.7
34	VolkWagen	GOLF GTE	55,7
35	ZERO MOTORCYCLES	DS ZF9	13
36	ZERO MOTORCYCLES	S ZF9	15,3
37		VOLTA BCN	3.1
38		Passat PHEV	9.9
39		XC90 T8 TWIN ENGINE	11.6
40	Audi	A3 E-TRON	8.8
41	Audi	A3 SPORTBACK E-TRON	8.8
42	BMW	225xe	7.6
43	BMW	225xe ACTIVE TOURER	10
44	BMW	225xe iPerformance	10
45	BMW	330e	12
46	BMW	C-EVOLUTION	8.1
47	BMW	E-EVOLUTION	8.1
48	BMW	i3	42.2
49	BMW	i3 REX	42.2
50	BMW	i8	7.1
51	BYD	E6	82
52	Citroen	C-ZERO	14.5
53	Citroen	C-ZERO SEDUCTION	16
54	Citroen	N. BERLINGO	22.5
55	HYUNDAI	IONIQ	28
56	HYUNDAI	KONA	64
57	Jaguar	I-PACE	90
58	KIA	NIRO PHEV	8.9
59	KIA	OPTIMA	10
60	KIA	SOUL EV	30
61	LEMEV	STREAM	4
62	MERCEDES-BENZ	GLC 350 E 4MATIC	13.5
63	MINI	COUNTRYMAN	7.6

We tried analysing the correlation between charge duration and the fraction of energy delivered with respect to the battery size of these EVs, which is a measure of the change in the SOC.

**Figure 10.**
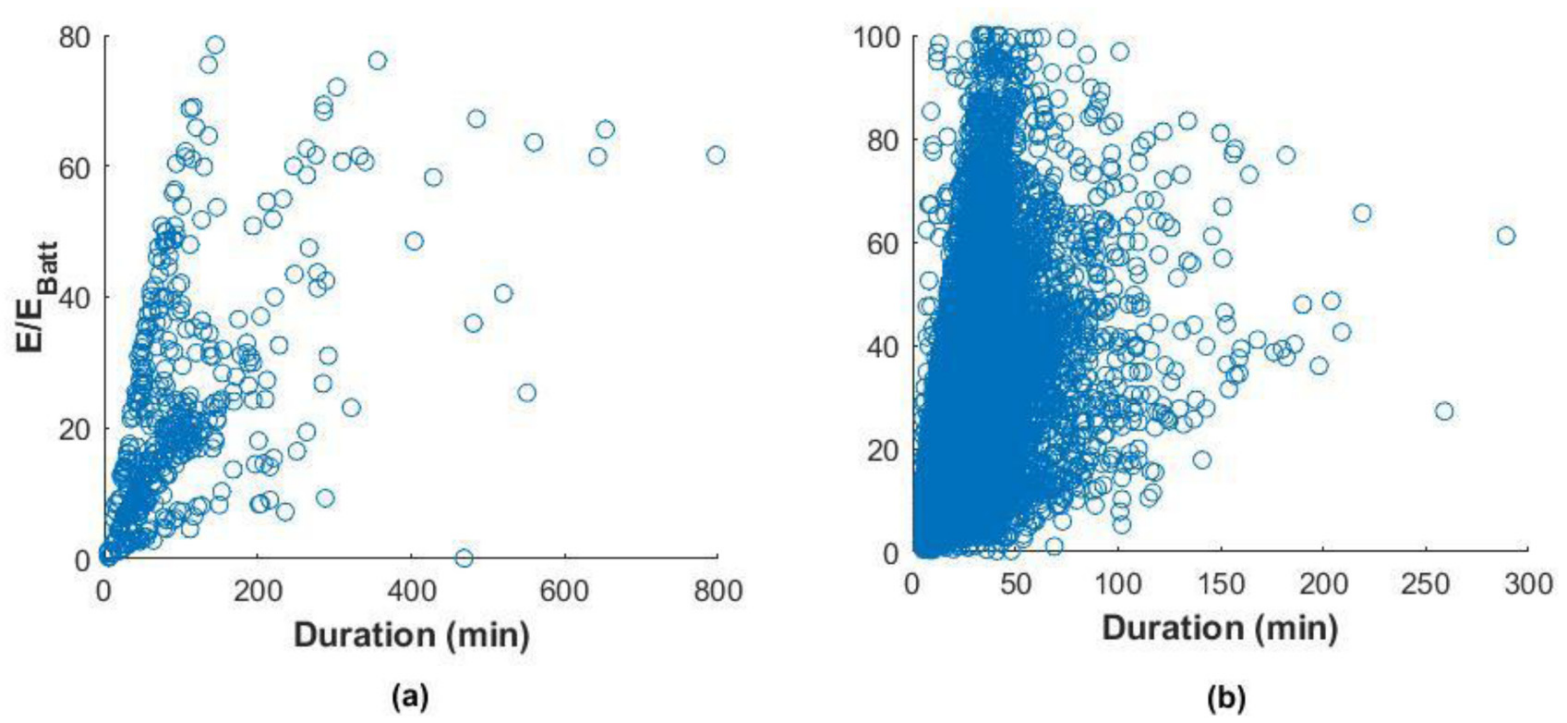
Correlation between the fraction of energy delivered and the charge duration for slow charges ( **a**) and fast charges ( **b**).

Using the Pearson correlation coefficient, which is a measure of the linear dependence of two random variables ^29^, we found a correlation coefficient of 0.54 and 0.41 for the slow and fast charger datasets, respectively ( Figure 10). This indicated a weak correlation among these variables, especially for fast charges.

**Figure 11.**
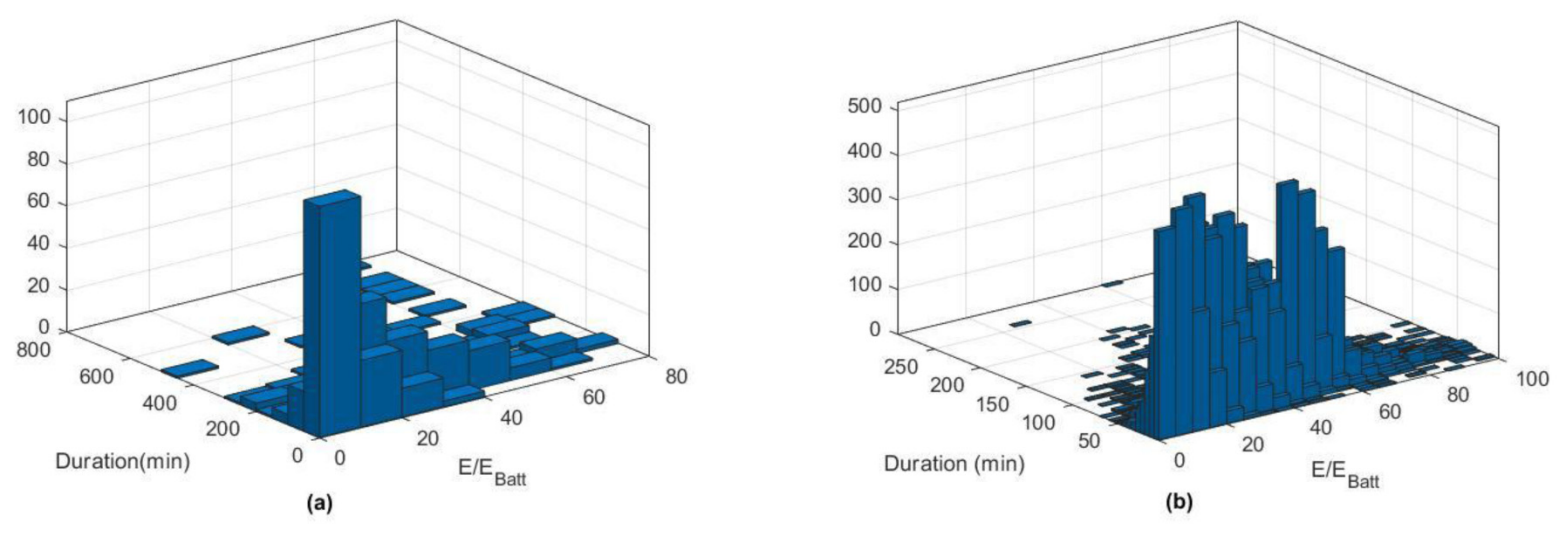
Slow ( **a**) and fast ( **b**) charge distribution as a function of charge duration and fraction of energy delivered.

This weak correlation can depend on different factors. For slow charges, a possible explanation would be a habit of leaving the car plugged after the charge is finished ^22^. For fast charges, the reason can be a tendency to charge even if the battery SOC is high or charge the battery until a SOC is close to 100%. In this case, the energy delivered is low, but the charging time could be longer since the charge is in the constant voltage phase, where the current is continually reduced to maintain a constant voltage. However, Figure 10(b) shows that most of the charges were within a duration range of fewer than 60 minutes. Indeed, analysis of the charge distributions showed that 73% of all the events charged less than 40% of the battery energy in less than 60 minutes. For slow charges, 80% filled less than 40% SOC in less than 200 minutes. Figure 11 reports the histograms for slow (a) and fast (b) charge distributions, as a function of the charge duration and the fraction of energy delivered with respect to the battery size.

### Turku dataset (Finland)

Data gathered for the city of Turku did not have information about the composition of the CSs. Moreover, a single physical address could be associated to more than one station ID. Therefore, we assumed that each CS was composed of a single CP and, one or more CPs corresponding to the same address, was considered as a CI. Consequently, we obtained:

- five CIs composed of two AC CPs;
- one CI composed of four AC CPs;
- one CI composed of one direct current (DC) CP;
- one CI composed of 2two AC CPs and one DC CP;

The power of the AC and DC CSs was not reported in the dataset, however, from city records, it was found to be 22 kW and 50 kW, respectively. The relevant statistical parameters for charging duration and energy delivered are presented in Table 7. As for the AMB dataset, DC charges (fast and ultrafast chargers) tended to be used more often than AC chargers.

**Table 7.** Mean values and standard deviations for different charge topology. CS: charging station; Stdev: Standard deviation.

Dataset	N charge/year/CS	N events/day/CS	Mean duration (min)	Stdev duration (min)	Mean energy (kWh)	Stdev energy (kWh)
Entire	363	1	195.31	502.38	6.31	6.64
AC	335.4	0.9	232.28	547.33	5.77	5.45
DC	584.5	1.6	25.58	25.88	8.80	10.14

**Figure 12.**
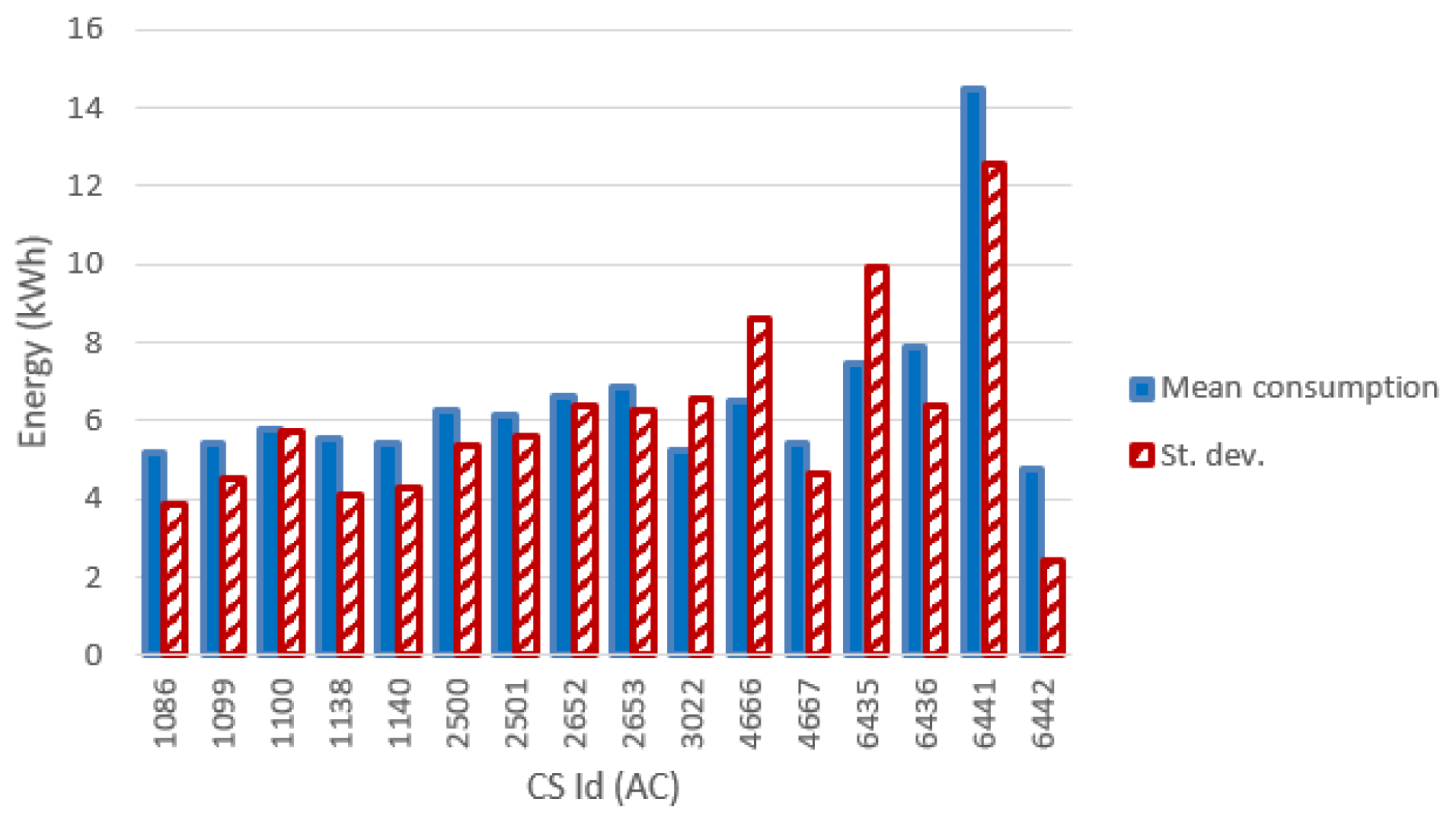
Mean value and standard deviation of the energy (in kWh) for alternative current (AC) chargers.

**Figure 13.**
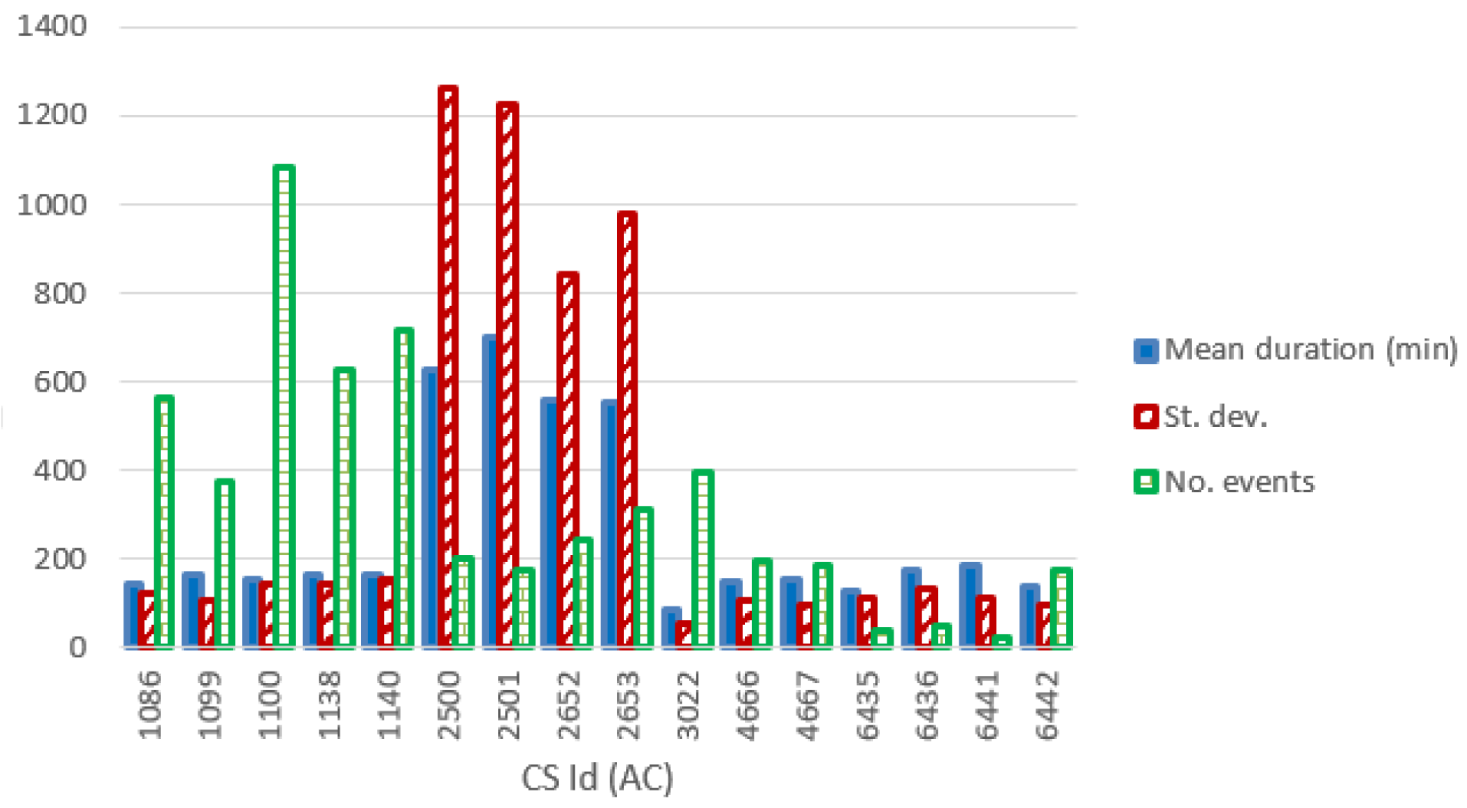
Number of charge events (yearly), mean value and standard deviation (St. dev.) of the duration (min) for alternative current (AC) chargers.

We could see a significant variability in the Turku dataset ( Table 5), especially for the AC charger where the standard deviation of the charge duration was quite remarkable compared to DC chargers. On the other hand, DC chargers had a non-negligible variability in the energy delivered compared to AC chargers. Figure 12 and Figure 13 show the most relevant statistical parameters for each AC CP, together with the number of events per year. Interesting, compared to the other CSs, the CS number 6441 delivered a considerable amount of energy ( Figure 12) but with the lowest usage rate ( Figure 13).

Data from the only two available DC CSs showed very similar values, both for the energy delivered and for the charge duration ( Table 8).

**Table 8.** Statistics for the two direct current (DC) charging points (CPs). Stdev: standard deviation.

DC CP ID	N charge/year	Mean duration (min)	Stdev duration (min)	Mean energy (kWh)	Stdev energy (kWh)
1126	480	21.8	15.0	9.2	8.5
3023	689	28.2	30.9	8.5	11.1

**Figure 14.**
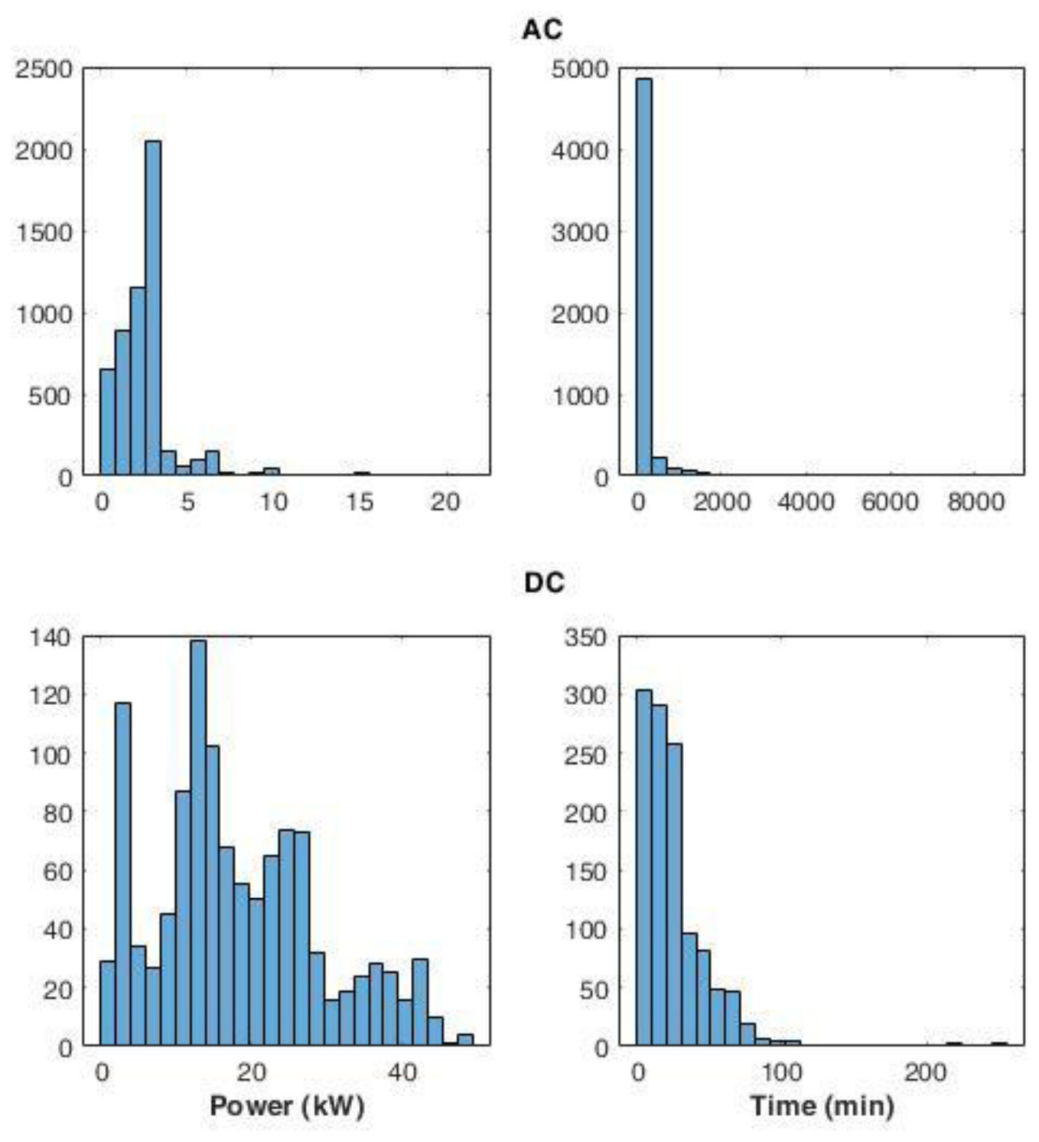
Distribution of the average power delivered during charging (left) and charge duration (right) at alternative current (AC) and direct current (DC) stations.

Figure 14 shows the distribution of the average power delivered during the charges (left panels), and the distribution for charge durations (right panels), for AC and DC chargers. The power distribution of AC charges showed that most of the charging events (99%) used less than half of the available power. The DC power distribution produced two main peaks: the most pronounced was observed between 10 and 15 kW, while a second peak was observed around 2 kW. Overall, 85% of the charge events had an average power lower than 30 kW.

AC and DC charge duration distributions showed a Poisson-like shape, with a long tail for the AC distribution. For AC, 92% of charge durations lasted between 0 and 400 minutes. On the other hand, for DC charges, 72% of the charge was achieved in less than 30 minutes.

**Figure 15.**
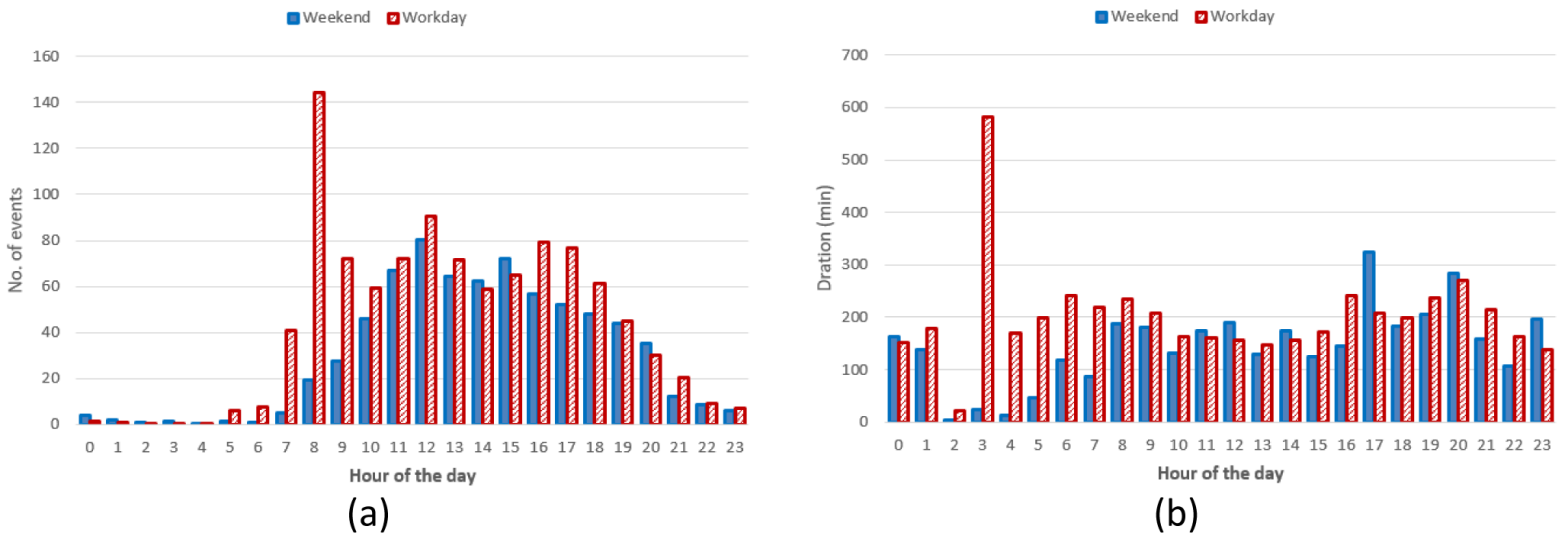
Distribution of the number of charges during the day ( **a**) and mean charge durations ( **b**).

***Daily and seasonal effects on charge distributions*.** As done for the AMB dataset, we analysed the hourly distribution of charging events. Figure 15(a) reports the hourly distribution of the number of charging events at their starting time (for both AC and DC), while Figure 15(b) shows their mean duration. Most charges started around 8 am during workdays, with a second peak at noon, followed by another local maximum around 4 pm for workdays ( Figure 15(a)). The duration of the charges started in the morning and late afternoon was longer, compared to the other times of the day ( Figure 15(b)).

**Figure 16.**
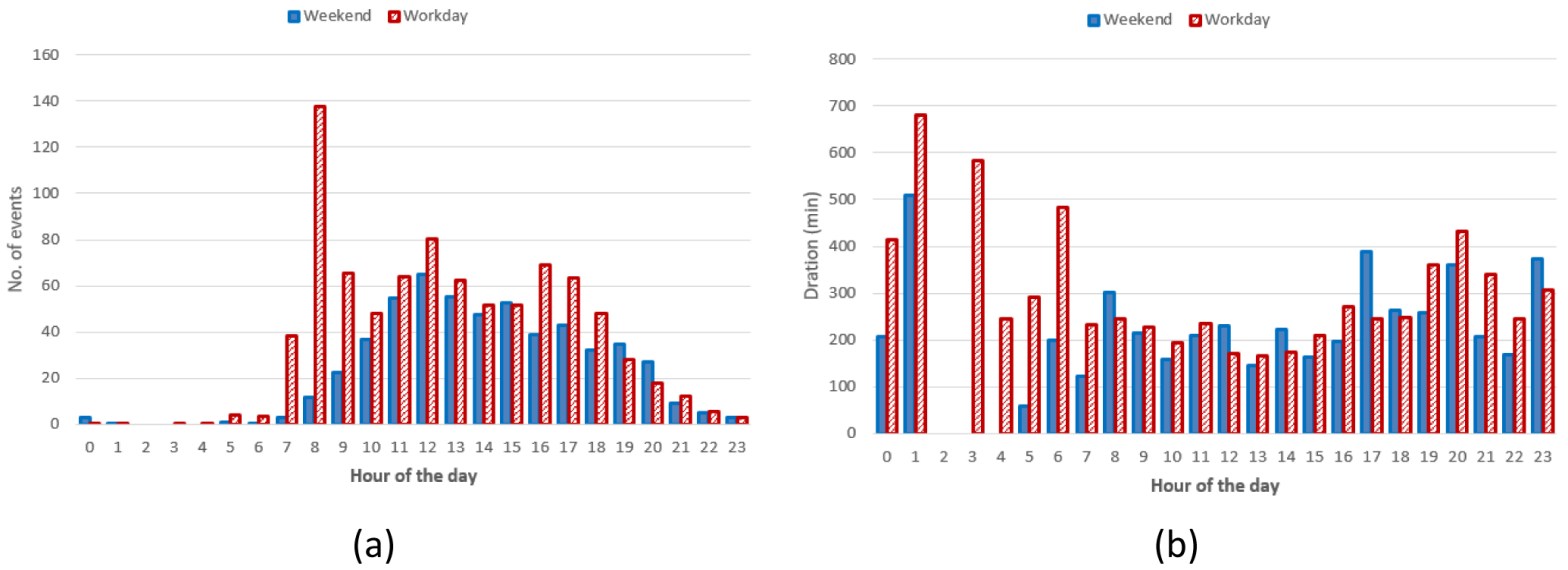
Distribution of number of charges ( **a**) and average duration ( **b**) during working days and weekends for alternative current (AC) charging points.

Hourly distribution of AC charges showed a remarkable high peak at 8 am ( Figure 16(a)) during working days but it disappeared during weekends. Other peaks observed during working days were around noon and 4 pm. Interestingly, during weekends, the majority of charges occurred around noon. On average, we could conclude the number of daily charges were higher during working days than during weekends. Similarly to the charge duration, the longest charging sessions were usually observed in the morning (although with a small number of occurrences) as well as in the afternoon ( Figure 16(b)).

**Figure 17.**
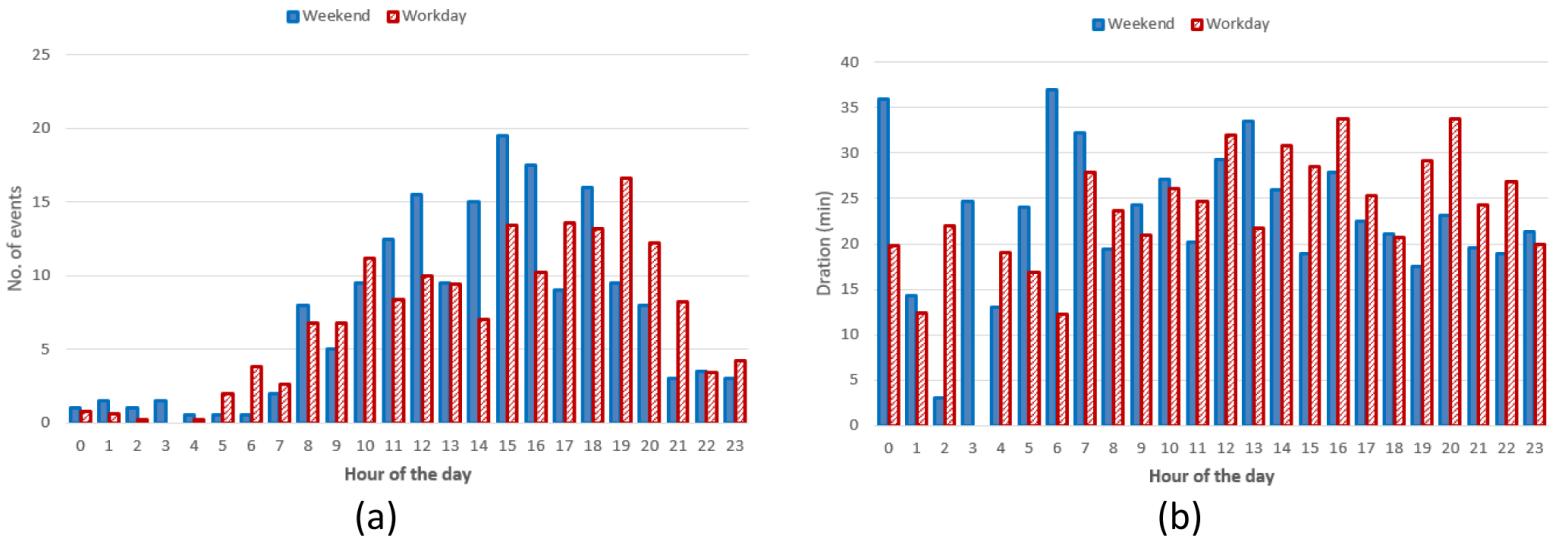
Distribution of number of charges ( **a**) and average duration ( **b**) during working days and weekends for direct current (DC) charging points (CPs).

Regarding the DC CSs, the number of hourly charges, was slightly higher during weekends than during working days ( Figure 17(a)). Moreover, DC charges were more frequent in the early afternoon during weekends and in the late afternoon during working days ( Figure 17(a)). Finally, the average charging duration did not show any particular pattern during working days and weekends ( Figure 17(b)).

Summary statistics for the Turku dataset ( Table 9) showed that AC charges were more frequent during weekends when they tended to be shorter and to deliver more energy compared to working days. On the other hand, DC charges were more frequent during working days when they delivered more energy and lasted longer.

**Table 9.** Mean values of charge duration and energy exchange for working days and weekends. AC: alternative current; DC: direct current; CS: charging station;

Dataset	N of events/day/CS Mon-Fri	N of events/ day/CS Sat & Sun	Mean duration Monday-Friday (min)	Mean duration Saturday and Sunday	Mean energy Monday-Friday (kWh)	Mean energy Saturday & Sunday (kWh)
Entire	0.8	1.1	199.98	178.72	6.21	6.67
AC	0.7	1.0	233.43	227.75	5.67	6.17
DC	1.7	1.6	26.36	23.69	9.02	8.27

**Figure 18.**
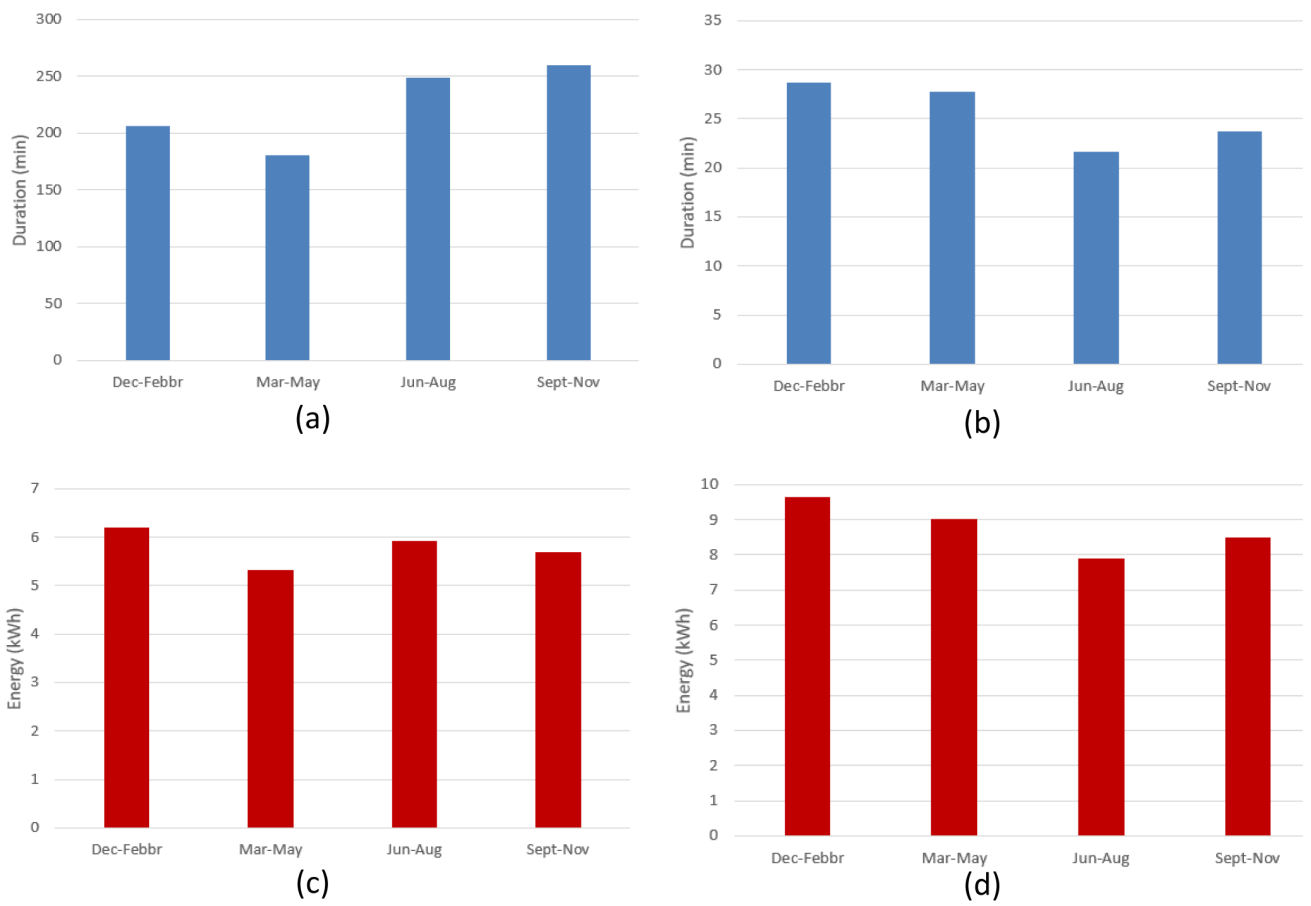
Seasonal influence on average charge parameters: ( **a**) alternative current (AC) charge duration; ( **b**) direct current (DC) charge duration; ( **c**) AC charge energy delivered; ( **d**) DC charge energy delivered.

Figure 18 illustrates the seasonal influence on charge duration and energy delivered. 

For AC charges, the difference between the longest duration (fall) and the shortest (spring) was around 44% ( Figure 18(a)), while the energy difference between the winter (highest value) and spring (lowest value) periods was around 14% ( Figure 18(c)). On the other hand, for DC charges, the shortest charging duration was observed in the summer period ( Figure 18(b)), and the difference with the longest charging duration (winter) was around 24% ( Figure 18(d)). Finally, the difference between the highest average energy delivered (winter) and the lowest one (summer) was around 18%. As for the AMB data, only the fast (DC) charge duration appear to follow the same seasonal trend as the delivered energy ( Figure 18(b), and Figure 18 (d)), with a correlation coefficient of about 0.88. However, no relevant correlation was observed for AC chargers.

### Simulation results

Simulation of the load demand at a CS was carried out using synthetic load profiles obtained from the statistical analysis of charge events. The fit of the charge registration data was used to build a nonparametric statistical distribution (NPD) of the frequency of hourly arrivals at the CSs for working days and weekends. Nonparametric estimations of the distribution function of data are not related to any specific *a priori* distribution and allow to generate random values that reproduce the observations closely. Among the nonparametric estimates, we chose a kernel density estimation (KDE) that creates a smooth curve from a dataset, from which inferences about the population can be made. The smoothness of the resulting curve depends on the bandwidth parameter. KDE was calculated by weighting the distances of all data from each value of the independent variable. A Gaussian kernel function was used and the KDE procedure was implemented with the Distribution Fitting Tool in Matlab software. An open-source alternative to perform the analysis is GNU Octave software ^30^. 

**Figure 19.**
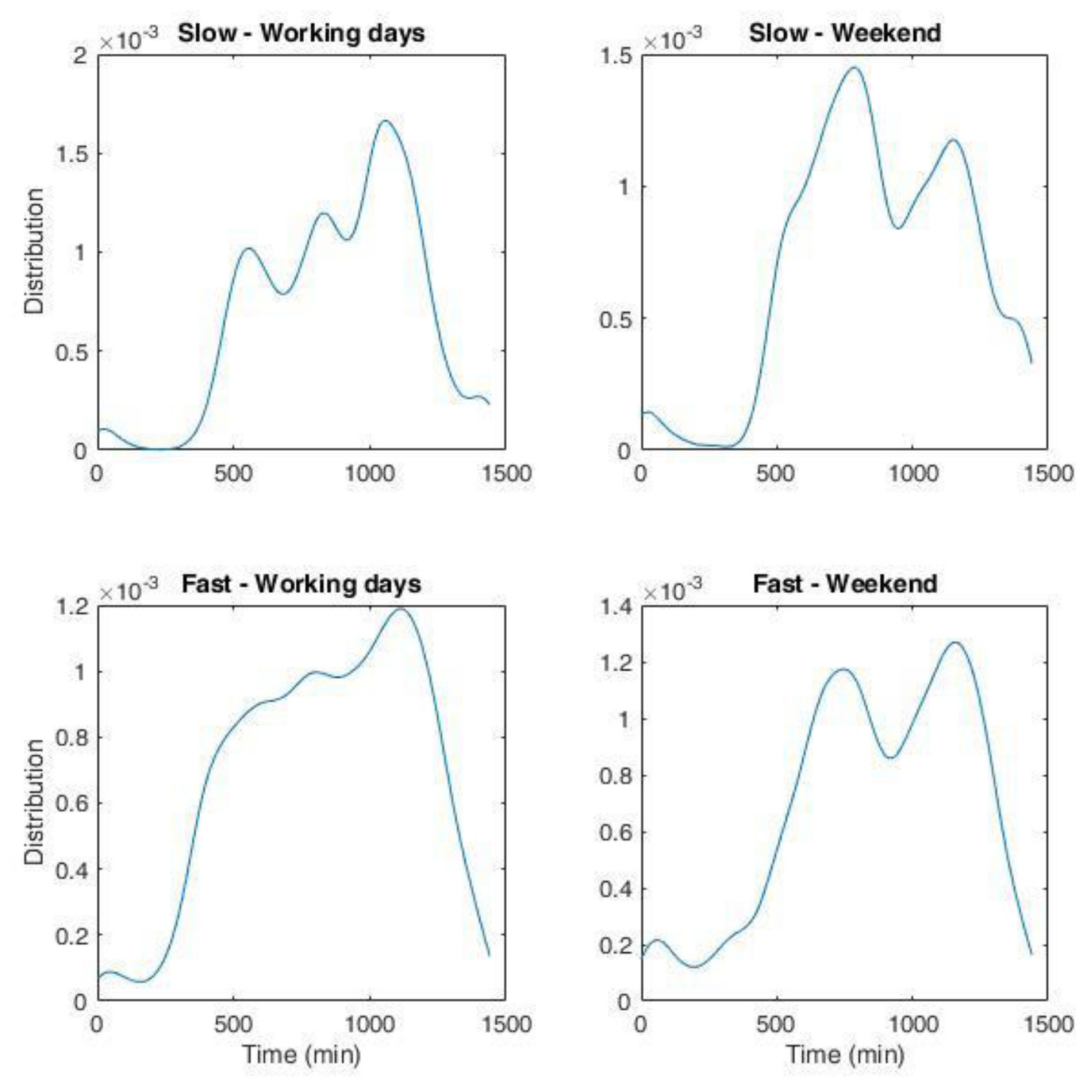
non-parametric distribution for the number of charges for the Area Metropolitana de Barcelona (AMB) dataset. Top left: slow charges during working days; top right: slow charges during weekend; bottom left: fast charges during working days; bottom right: fast charges during weekend. The whole duration in minutes corresponds to one day.

NPDs for slow and fast chargers during working days and weekends are shown in Figure 19. The bandwidth of the fit was set at 45 minutes.

**Figure 20.**
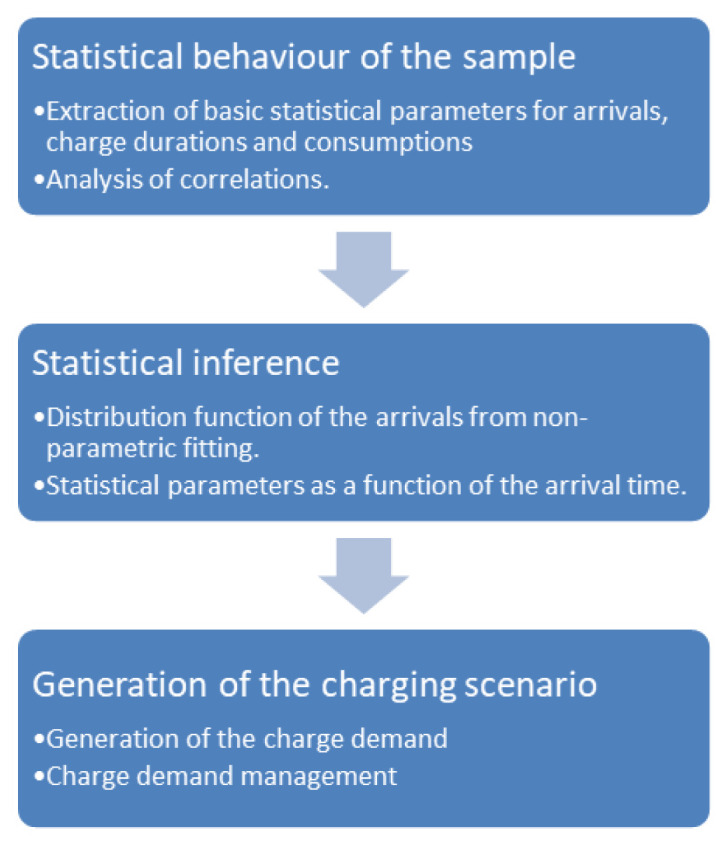
Synthetic profiles generation workflow.

We obtained the distribution of arrivals for a given number of users/day from the NPDs. Using the statistical parameters obtained from the analysis of the AMB dataset, such as the average energy and charge durations as a function of time and day, and their respective variances, we could generate several possible scenarios of charge demand to obtain information on different variables of interest for CI management, such as energy demand and queuing. The workflow is illustrated in Figure 20.

To determine the charge duration and the energy required, we made the hypothesis that both followed a Gaussian distribution with mean values obtained from the input data corresponding to their hourly trend during working days and weekends. Since we only considered positive values, the resulting distributions strongly resembled those reported in Figure 5. For the variance, we used the standard deviation of the input dataset. With this data, and the NPDs, we could determine a scenario of arrivals at a CS for a given average number of daily arrivals. To increase the variability of the data, we added a white noise signal to the charging duration and charging energy. In the following, we will refer to these profiles as “stochastic synthetic profiles”.

**Figure 21.**
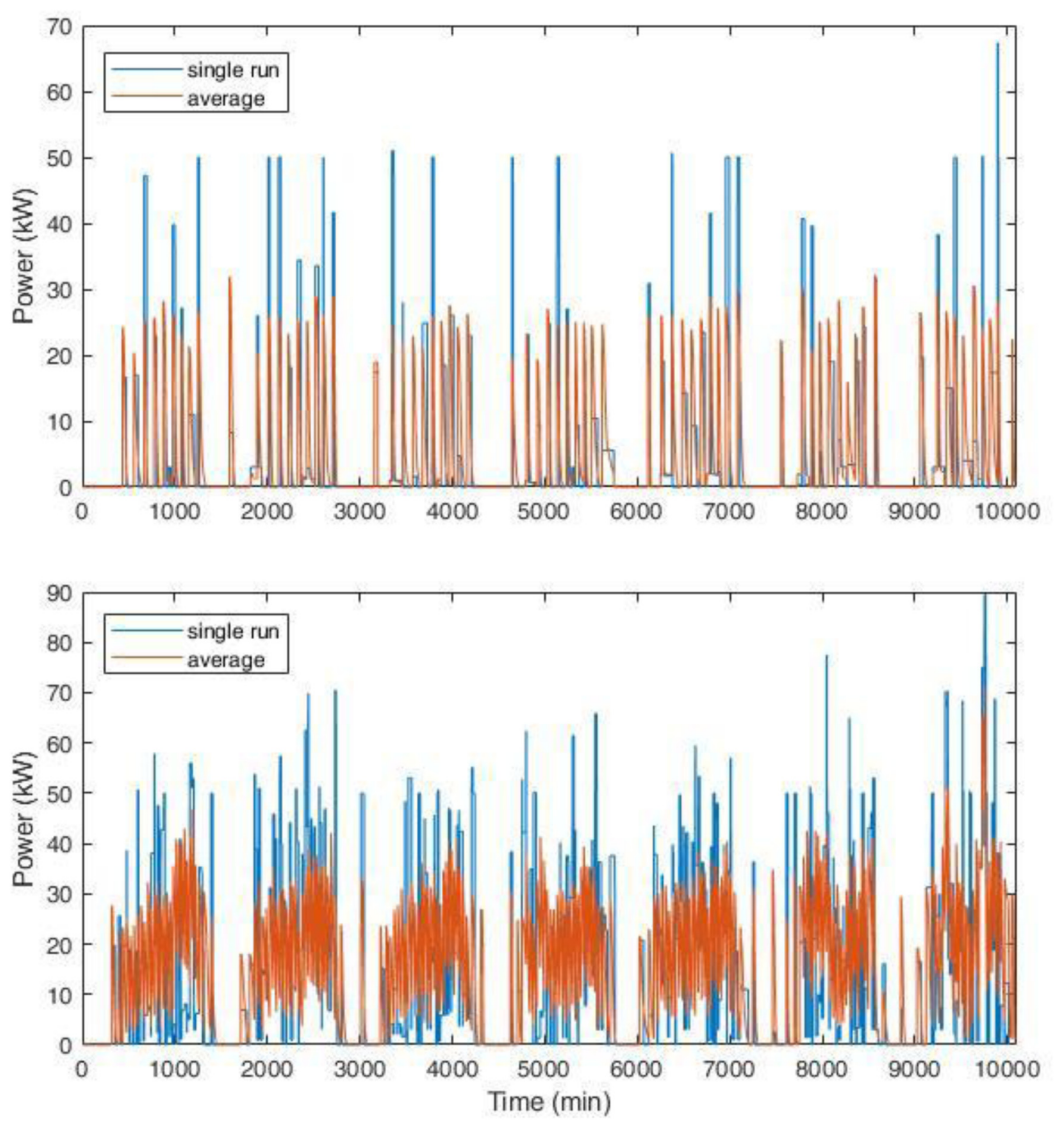
Synthetic load profiles for the actual user inflow (top, for an average of one and nine daily users for slow and fast charges, respectively) and for a projection with tripled influx (bottom, six users at slow charges and 30 at fast charges on average per day). The blue line represents the load for a single simulation, while the red line is the average load over 1000 runs for one week. The length of each profile is one week.

Two examples of the synthetic profiles used in the simulations are shown in Figure 21. Considering the typical CI for AMB, which was composed of two slow CPs (3 kW) and three fast CPs (50 kW), we compared the profile for a single run (one week) with the average profile over 1000 simulations. Figure 21 (top) shows the results for an average of one and nine daily users for slow and fast charges, respectively. The choice of these values was in line with the AMB statistics reported in Table 2. The bottom of Figure 21 shows the results for a tripled number of daily users at the CI (six users at slow charges and 30 at fast charges on average per day).

Average data shown in Figure 21 smoothed the peaks and distributed the charging requests more evenly. This could affect the evaluation of the CI size, especially if auxiliary services were present, such as a renewable energy source and storage systems.

To check the impact of the profiles used as input in sizing problems, we applied an optimization procedure to determine the size of a photovoltaic (PV) system to be installed in a CI according to different load profiles. For this purpose, we used the economic criterion of the net present value (NPV) which allowed to compare the advantage of an investment over another scenario. NPV is expressed as follows:


$$NPV=\sum_{t=1}^{N}\frac{F_{t}}{{\left(1+k\right)}^{t}}-I\;(1)$$

where:

*N* is the time horizon of the investment in years; *F _t_* is the cash flows in the t ^th^ year, calculated as the difference between cash flow without and with the PV system; *I* is the initial investment for the PV system; *k* is the interest rate fixed at 3%. A positive NPV means the investment is convenient. The analysis was carried out over the entire depreciation period of the infrastructure, which was assumed to be of 20 years, i.e., the medium PV life. In this approach, the PV size is determined according to the charging profiles, grid energy costs, device investment and operational costs. The analysis was proposed in 31 for a PV and storage system. However, in this work we only report the simplified version without the storage.

The optimization procedure consisted in minimizing the objective function represented by the daily operating cost of the system, which in this case was only the cost of the energy supplied by the grid. The operational cost of the PV was assumed to be negligible, while the degradation was considered. The formal expression of the optimization problem is as follows:


$$\min C_{e}=\min\sum_{h=1}^{24}\left(C_{r}(h)\;P_{grid}(h)\text{Δ}t\right)+C_{degr}\;(2)$$

where:

*C _e_* is the daily cost [€]; *C _r_* ( *h*) is the price of the energy at time *h* [€/kWh];

*C _degr_* is the degradation cost of the PV system [€/day];

*P _grid_* ( *h*) is the power withdrawn from the grid at time *h* [W];

Δ *t* is the sample time, which in this analysis was equal to one hour.

The model of the system must respect a series of constraints listed below:


$$P_{grid}(h)\eta_{grid}+P_{PV}(h)\eta_{PV}=\frac{P_{L(h)}}{\eta_{L}}\;(3)$$

$$P_{grid}^{min}\leq P_{grid}(h)\leq P_{grid}^{Max}\;(4)$$

$$P_{PV}^{min}\leq P_{PV}(h)\leq P_{PV}^{Max}\;(5)$$

$$P_{PV}(h)\leq P_{mppt}(h)\;(6)$$

where:

*P _grid_* is the power withdrawn from the grid;

*P _PV_* is the power supplied by the photovoltaic system;

*P _L_* is the power required by the load;

P _mppt_ is the maximum extractable PV power;

*η _grid_* is the efficiency of the network converter;

*η _PV_* is the efficiency of the photovoltaic system converter;

*η _L_* is the efficiency of the output converter.

Equation (3) represents the power balance of the system and takes into account the efficiencies to ensure the charging power required by the load at the h ^th^ hour. Equation (4) and Equation (5) define the operating limits of the system based on the minimum and maximum power of the sources. Equation (6) limits the power that can be drawn from the photovoltaic system to the maximum extractable power P _mppt_ in the h ^th^ hour.

The optimization procedure inputs were as follows:

a) Price of electricity: 2019 data were retrieved from the Comisión Nacional de los Mercados y la Competencia (CNMC) website ^32^;
b) Cost of the photovoltaic system: 1.2 €/Wp and 0.019 €/Wp, for the capital expenditure (CAPEX), namely the investment costs, and the operating expenditure (OPEX) to run the system ^33, 34^;
c) Productivity of the PV system: data were taken from the “Performance of grid-connected PV” tool of the Photovoltaic Geographical Information System ( PVGIS) ^35^. The data contained the monthly production for an installed peak PV power of 1 kWp and system loss of 14% (mounting configuration: slope 35⁰, azimuth 0⁰) from the PVGIS-SARAH database, for the selected locations.
d) Efficiencies of the conversion systems: some new solutions showed efficiencies higher than 0.9 ^31^. However, efficiency values of 0.9 were used for all systems.

We used the average annual values for electricity and insulation prices. For the charging power profiles, we used different statistic approaches, to compare the results and determine the optimal sizing, and to understand possible differences. In all cases, the power load profile was extracted from the dataset of charging events. The profiles used in the procedure were the following:

1. The first profile was obtained using the average values of the energy and charge durations for each time step, in minutes, to build the average power profile weighted by the frequency distribution of arrivals.
2. The second profile was the average of 5,000 synthetic load profiles.
3. The third profile was obtained with the same procedure as profile ‎2 using mode values instead of mean values.

**Figure 22.**
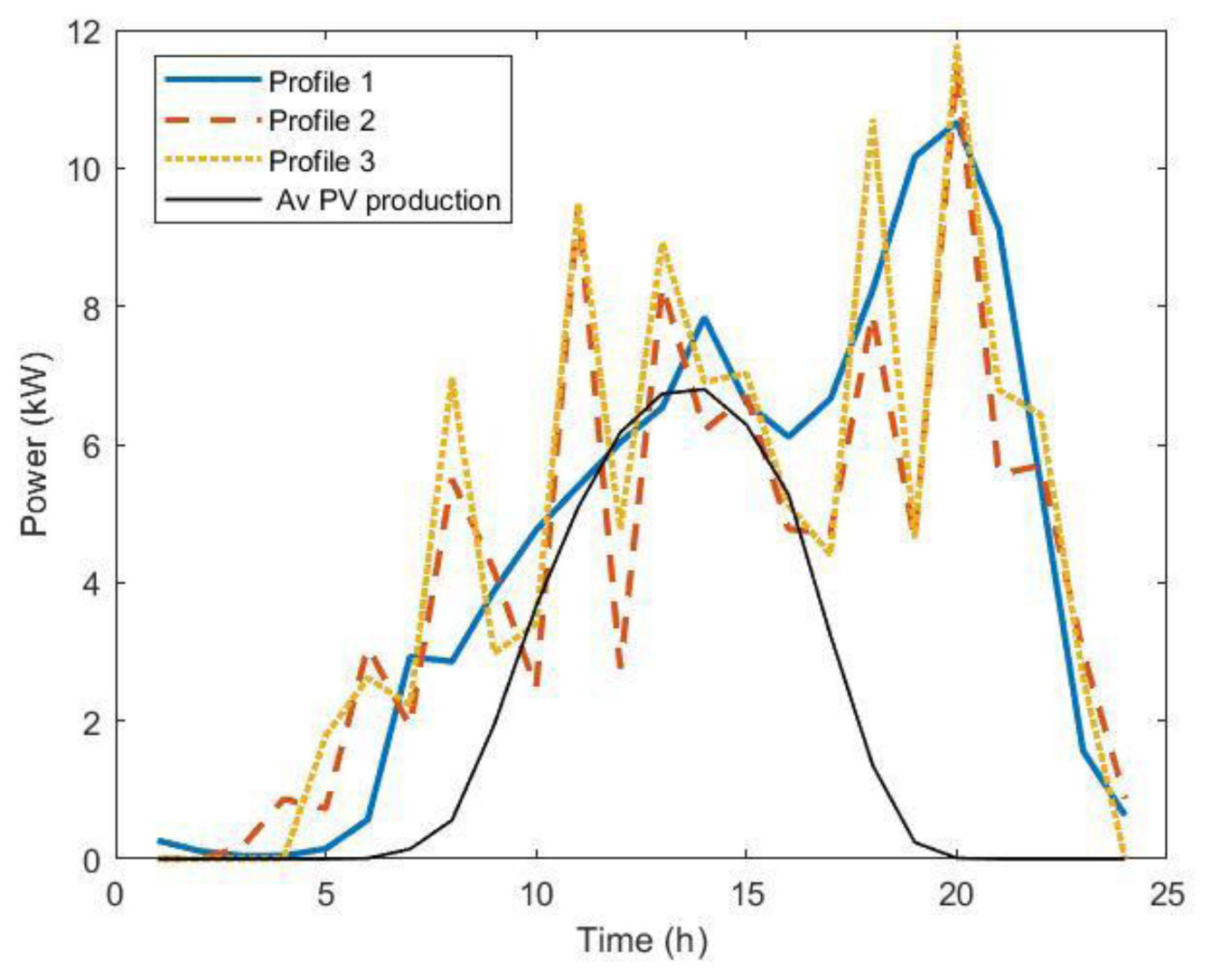
The three load profiles used in the optimization procedure. Profile 1: average over dataset (blue solid line); Profile 2: average over 5,000 synthetic load profiles; Profile 3: modal value over 5,000 synthetic load profiles. The black line is the average annual power production for 11 kW photovoltaic systems (PV).

For profiles 2 and 3, we used an average occupancy of one user/day for slow chargers and nine users/day for fast chargers. The three profiles are shown in Figure 22. Profiles 2 and 3 showed a similar intermittent pattern, while profile 1 was smoother. Power peaks were more pronounced for profile 3.

Optimal PV size results obtained through this procedure using the three different profiles were 13.2 KW, 10.9 kW and 11.3 kW for profile 1, 2, and 3, respectively. We observed that using the average values of the entire AMB dataset gave a slightly larger optimal size for the PV compared to the other load profiles. Considering the average annual power production for an 11kW PV system (black line, Figure 22), we can see that for profile 1, there were fewer time intervals when the load was lower than the PV production, i.e., time intervals for which PV production did not contribute to cost saving. In fact, in the analysis we did not consider selling PV energy to the grid.

To verify which solutions was best for actual usage, we considered the power profile of the charge registrations. We then calculated the NPV for CI+PV systems, with the hypothesis that the load remained the same for the whole depreciation period. For this purpose, we selected the most and least busy CSs to build two hypothetical CIs according to these two scenarios ( Table 10).

**Table 10.** Information on the most and less crowded charging stations (CSs) in the Area Metropolitana de Barcelona (AMB) database.

CS	ID fast	ID slow	User/year fast	User/year slow
Most crowded	16	6	5121	616
Less crowded	18	3	1378	55

We assessed the NPV for the most and least crowded CSs, using the PV sizes obtained from the optimization procedures with profiles 1 to 3 and reported the results in Table 11.

**Table 11.** Net present value (NPV, in k€) for two different photovoltaic system PV sizes in the most and least crowded charging infrastructures (CIs) scenarios.

PV size (kW)	NPV most crowded CI (k€)	NPV least crowded CI (k€)
13.5 (profile 1)	-4.4	-12.7
10.5 (profile 2)	-3.1	-9.7
11.3 (profile 3)	-3.5	-10.5

Results showed that NPV was negative in all cases, meaning the PV sizes obtained using the proposed profiles were not convenient ( Table 11). On average, only 70% and 20% of the PV energy was used to match the power demand in the most and least crowded cases, respectively. This means that optimal PV sizes for the average profiles were overestimated for real profiles. We thus evaluated the optimal PV size using a different method involving an MC workflow. In this approach, we created a stochastic synthetic profile, estimated the optimal PV size using NPV criteria, and repeated the procedure N times, where N ranged from 10 up to 1,500. Results were then compared for different number of runs. Finally, we averaged all the PV values obtained from all runs. We assumed an influx of one user/day at slow CPs and nine users/day at fast CPs in a typical CI (two slow CPs and three fast ones) to create synthetic profiles as those in Figure 21 (top panel). To simulate the uncertainties on the arrivals, we added white noise to the NPDs of Figure 19.

**Figure 23.**
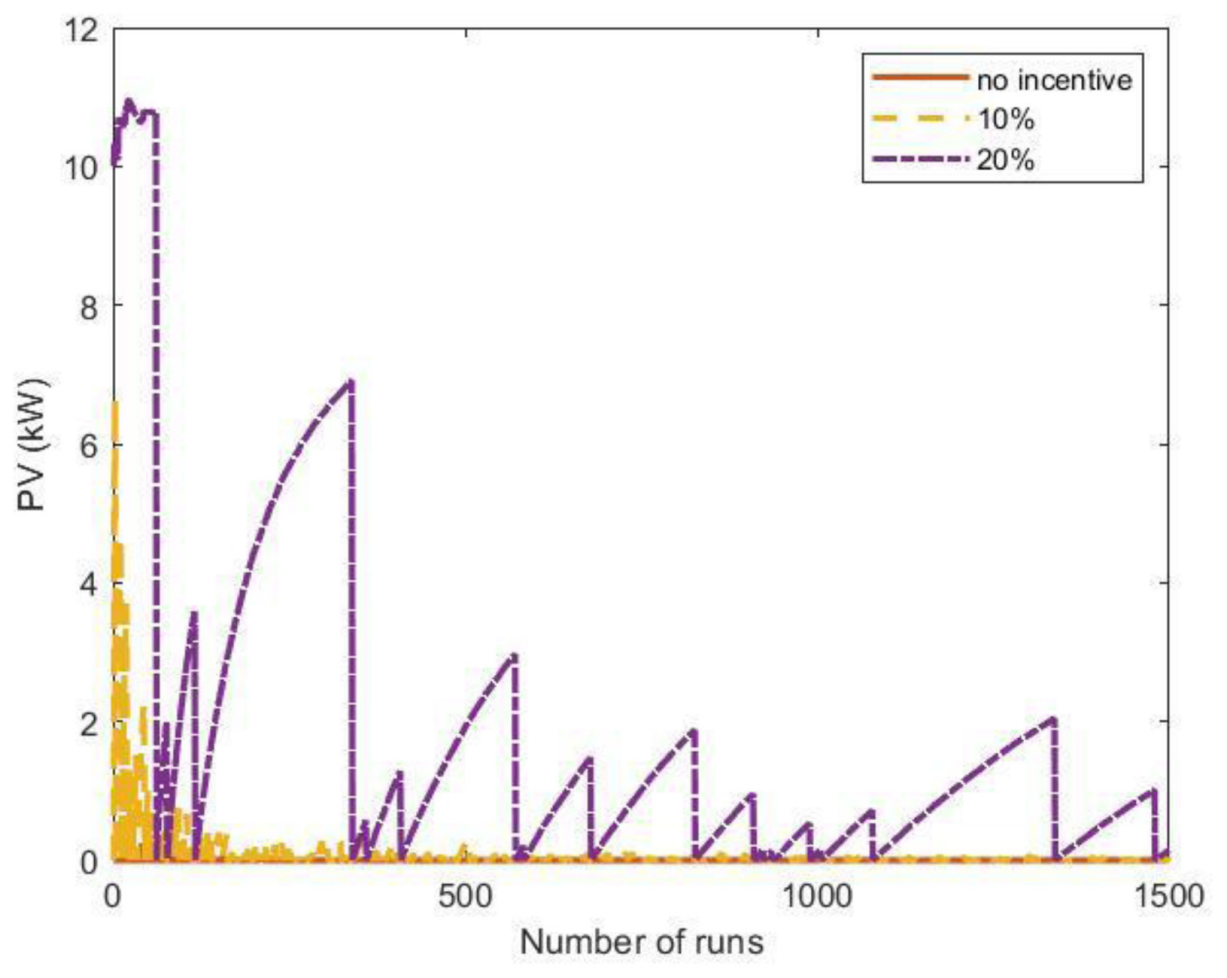
Optimal photovoltaic system (PV) size as a function of the number of Monte Carlo simulation runs using synthetic profiles with white noise. Three scenarios were analysed: no financial incentives on the initial investment (solid red line); 10% discount incentives (yellow dashed line); 20% discount incentives (purple dashed-dotted line).

Figure 23 shows the results of the MC procedure for the optimal PV size as a function of the number of runs. The red line represents the average value and converges rapidly to zero, meaning that investing in a PV system was not convenient. We also included the results when some financial incentives policies for the initial investment of the PV system were considered. Possible financial incentives were at governmental level on the CAPEX of the PV system. Considering a 10% discount on the initial PV cost, the PV system remained an unfavourable solution (yellow dashed line). With a 20% incentive, the solution appeared to converge to zero, although with many fluctuations. 

When the fluctuations on the arrival distributions were not included in the MC simulation, the results changed dramatically. When the synthetic profiles were generated without adding white noise, the outcomes of the MC simulations converged to a solution for the PV size greater than zero if incentives were included.

**Figure 24.**
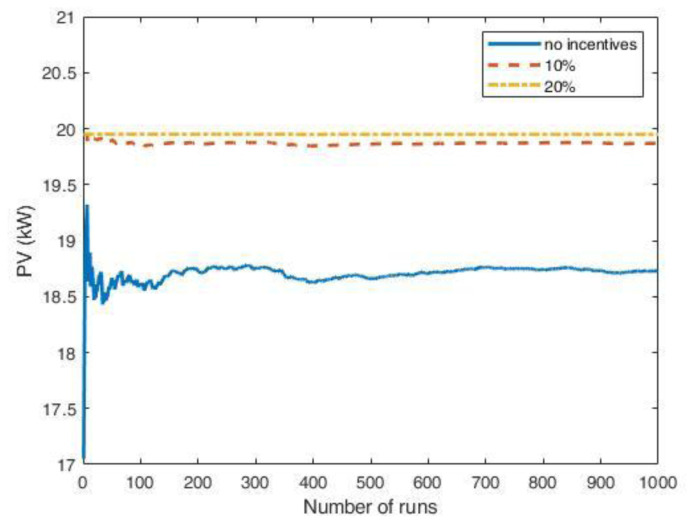
Optimal PV size as a function of the number of Monte Carlo simulation runs using synthetic profiles with white noise, for an average influx of 18 users at fast CSs and two users at slow CSs per day. The scenarios include: no financial incentives on the initial investment (solid blue line); 10% discount incentives (red dashed line); 20% discount incentives (yellow dashed-dotted line).

The influence of the arrival fluctuations tended to disappear when the influx rate increased. As an example, Figure 24 reports the results of a MC simulation of the PV size, where the synthetic profiles referred to an average influx of 18 users at fast CSs and two users at slow CSs, and included white noise. In that case, the simulations showed that the PV system was convenient, even without financial incentives.

We conclude that synthetic load profiles are convenient tools for analysing different scenarios of electric mobility spread. However, when the usage rate of the charging infrastructure is low, data fluctuation must be considered, as it might heavily affect the load profiles.

## Conclusions

In this study, we analysed two EV charging events datasets, for the metropolitan area of Barcelona (Spain) and the city of Turku (Finland) at public stations. Charging events referred to different charge modes, such as slow (3 kW, 7 kW, 22 kW) and fast (43 kW, 50 kW, 55 kW) chargers. The statistical analysis revealed some distinct features for fast and slow charges. Specifically, the average fast charge duration was around 27 minutes for both datasets, with an average delivered energy of 10 kWh. Slow charges showed a much higher duration (around three hours) and less energy delivered (around 5 kWh). Moreover, slow charges showed a greater standard deviation in the duration distribution than fast charges, while the energy distributions had comparable standard deviations. Time and energy dispersions were higher for the Turku dataset than for AMB, likely because the Turku dataset was smaller than the AMB dataset. Different patterns emerged in the distributions of charging start times for fast and slow charges, with distinguishable features depending on the data set under consideration. For the AMB dataset, fast charge starting hours were relatively homogeneously distributed during daytime and early evening, while two peaks were visible in the distribution of slow charges starting times. For the Turku dataset, AC charges started predominantly during night-time and early morning, while DC charges started prevalently during daytime and late evening.

Statistical analysis of EV charge data might represent the starting point for inferring users’ profiles at charging stations for different mobility scenarios. Different synthetic profiles obtained in this work were used as inputs for an MC approach to determine the energy flow at a typical charging station. Analysis of energy flows at charging stations allowed the evaluation of the impact of different charge profiles to determine the optimal size of a PV system for a charging station. The NPV economic criterion used to determine the size of the PV system showed that overlooking fluctuations in charging profiles could lead to overestimating the optimal PV size.

## Data availability

### Underlying data

Zenodo: Support data for "Modelling charge profiles of electric vehicles based on charges data". https://doi.org/10.5281/zenodo.5721233 ^28^.

This project contains the following underlying data:

- HISTORIC DATA 2019 ELECTROLINERES AMB.csv: contains information on the charge events at the public charging points managed by the municipality in the metropolitan area of Barcelona. Fields are: charging point name; connector typology and number; charge start time; charge stop time; charge duration in minutes, energy delivered in kWh; vehicle manufacturer (optional); vehicle model (optional).
- STATIC INFORMATION CHARGING POINTS AMB 29042020.csv: contains the information about the public charging points of the metropolitan area of Barcelona. Fields are: charger typology (Quick/Normal); Charging point name and address; OCCP version; charger location; longitude; latitude; 7 flag fields for the connector type; observations; charging point maker.
- Lataustapahtumat, julkiset latauslaitteet 2019.csv: contains information on the charge events at the public charging points in the city of Turku. Fields are: data of record creation; charging station Id: charging station name; charge start time; charge stop time; charge duration in minutes, energy delivered in Wh; plug type (AC/DC); cumulative energy of the dataset in Wh; average power of the charge in W.

### Extended data

Zenodo: Support data for "Modelling charge profiles of electric vehicles based on charges data". https://doi.org/10.5281/zenodo.5721233 ^28^.

This project contains the following extended data:

- EV.csv: contains data on battery size retrieved from vehicle data sheet or manufacturer website. Fields are: record ID, vehicle manufacturer; vehicle model; battery size in kWh.
- Charge2019_EV_AMB.csv: contains the data on charge requests contained in “HISTORIC DATA 2019 ELECTROLINERES AMB.csv” file, combined with the information on vehicle battery contained in “EV.csv” file.

Data are available under the terms of the Creative Commons Attribution 4.0 International license (CC-BY 4.0).
