# Supplementary material for: Modelling charge profiles of electric vehicles based on charges data
Source: Open Res Eur. 2022 May 25;1:156. Originally published 2021 Dec 23. [Version 3] doi: 10.12688/openreseurope.14354.3 (PMC10446039; doi:10.12688/openreseurope.14354.3)
Supplement: Peer review history

# Peer review: Reviewer response for version 2

**DOI:** 10.21956/openreseurope.15672.r28575

**Published:** 2022-03-14

**Authors:** Sandford Bessler

**Affiliations:** 1 TU Wien, Wien, Austria

My only comment has to do with structuring the paper in sections to improve the readability. Section Results and Discussion for example starts with page 5 and ends on page 26. In my view, there are at least 3 topics discussed there: 1. analysis of the charging data, 2. generation of charging load profiles and  3. (page 22 bottom) use of charging load profiles for planning an additional PV system.

 After these minor changes, I think the paper status should be accepted.

Is the study design appropriate and does the work have academic merit?

Yes

Is the work clearly and accurately presented and does it cite the current literature?

Partly

If applicable, is the statistical analysis and its interpretation appropriate?

I cannot comment. A qualified statistician is required.

Are all the source data underlying the results available to ensure full reproducibility?

Yes

Are the conclusions drawn adequately supported by the results?

Yes

Are sufficient details of methods and analysis provided to allow replication by others?

Partly

Reviewer Expertise:

EV charging optimization, smart grids, renewable energy communities

I confirm that I have read this submission and believe that I have an appropriate level of expertise to confirm that it is of an acceptable scientific standard.

# Peer review: Reviewer response for version 2

**DOI:** 10.21956/openreseurope.15672.r28574

**Published:** 2022-02-16

**Authors:** Tiago Pinto

**Affiliations:** 1 Research Group on Intelligent Engineering and Computing for Advanced Innovation and Development (GECAD), School of Engineering, Polytechnic of Porto (ISEP/IPP), Porto, Portugal

The article has been revised well, addressing the review comments in a suitable way. I recommend that this article pass peer review.

Is the study design appropriate and does the work have academic merit?

Yes

Is the work clearly and accurately presented and does it cite the current literature?

Partly

If applicable, is the statistical analysis and its interpretation appropriate?

I cannot comment. A qualified statistician is required.

Are all the source data underlying the results available to ensure full reproducibility?

Yes

Are the conclusions drawn adequately supported by the results?

Yes

Are sufficient details of methods and analysis provided to allow replication by others?

Partly

Reviewer Expertise:

electricity markets, renewable energy sources, artificial intelligence

I confirm that I have read this submission and believe that I have an appropriate level of expertise to confirm that it is of an acceptable scientific standard.

# Peer review: Reviewer response for version 1

**DOI:** 10.21956/openreseurope.15491.r28230

**Published:** 2022-01-25

**Authors:** Tiago Pinto

**Affiliations:** 1 Research Group on Intelligent Engineering and Computing for Advanced Innovation and Development (GECAD), School of Engineering, Polytechnic of Porto (ISEP/IPP), Porto, Portugal

This paper presents an analysis of data from two cities, regarding energy and duration required to charge Electric Vehicles (EV). The study focuses on hourly, daily and seasonal patterns in charge duration and energy delivered. Simulated scenarios for the power request at charging stations are created using statistical parameters and non-parametric distributions of the arrivals. Different scenarios of users’ influx at the charging stations are defined using Monte Carlo simulations, which are then used to determine the optimal size of a PV system. Results show that distinct features for the cases of slow or fast charges have emerged in the hourly distribution of the requests. The investment and optimal size of the PV system vary substantially according to the different charging profiles.

 Overall, the paper addresses a timely and relevant research topic and introduces a good contextualization of the work as well as a complete set of statistical analyses on the considered datasets. However, there are several issues that should be addressed in order to make this work clear to the reader, and easier to follow and extract significant conclusions from the work:

- Abstract: Electric Vehicles (EV) are introduced twice.
- The introduction provides a nice review of related work. However, it fails to clearly identify the gaps in current literature and thus motivate the original contributions of this work. At the end of the introduction, the reader should already be well aware of why such a statistical analysis is needed, as well as the application of Monte Carlo simulations and synthetic charging profiles generation, so that one may reach a suitable estimation of the requirements and investment needed to support such a charging infrastructure.
- The Monte Carlo simulation process should be detailed, as besides the achieved results, there is nearly no information on how the process has been performed.
- The overall organization of the document does not help the reader, in specific, the places where figures and tables are presented are often highly disconnected with the pieces of text that discuss them. This makes the analysis of such a large number of analyses even more difficult.
- The results on the potential advantages and sizing of the PV system should be more clearly discussed.
- The reference list is adequate.

Is the study design appropriate and does the work have academic merit?

Yes

Is the work clearly and accurately presented and does it cite the current literature?

Partly

If applicable, is the statistical analysis and its interpretation appropriate?

I cannot comment. A qualified statistician is required.

Are all the source data underlying the results available to ensure full reproducibility?

Yes

Are the conclusions drawn adequately supported by the results?

Yes

Are sufficient details of methods and analysis provided to allow replication by others?

Partly

Reviewer Expertise:

electricity markets, renewable energy sources, artificial intelligence

I confirm that I have read this submission and believe that I have an appropriate level of expertise to confirm that it is of an acceptable scientific standard, however I have significant reservations, as outlined above.

# Response

**Published:** 2022-02-04

**Authors:** Natascia Andrenacci

**Affiliations:** Ente per le Nuove Tecnologie, l'Energia e l'Ambiente (ENEA), Italy

*The authors would like to thank the reviewer for his comments and valuable input.* *In the following, we have tried to respond to the observations point by point. Our responses are shown in italics, and changes made to the text of the article are underlined.*

- Abstract: Electric Vehicles (EV) are introduced twice.
*Thank you for pointing out the mistake. We fixed it.*
- The introduction provides a nice review of related work. However, it fails to clearly identify the gaps in current literature and thus motivate the original contributions of this work. At the end of the introduction, the reader should already be well aware of why such a statistical analysis is needed, as well as the application of Monte Carlo simulations and synthetic charging profiles generation, so that one may reach a suitable estimation of the requirements and investment needed to support such a charging infrastructure.
*Thank you for the valuable comment. We added the following sentence at the end of the Introduction: “ The study of the charging profiles and the potential energy needs of electric vehicles is used for the correct design and planning of the activities of the charging structures. As far as the authors know, the impact of the different methodologies used to obtain these profiles on the optimization process has not been investigated. The present work aims to underline the impact that charging profiles obtained with different methods can have on determining the optimal investment for a renewable source implemented in charging infrastructure. We conducted statistical analysis on the data collected in some public recharging points in the cities of Barcelona and Turku. The results are the input to synthesize charging demand profiles according to different approaches. We compared the optimal PV sizes obtained with these different load profiles, highlighting that statistical approaches based on the measures of central tendency are not suitable for low usage rates of the charging infrastructures. Non-parametric statistical distribution (NPD)* *20* *combined with a Monte Carlo approach can provide more adequate results for this situation* *.”*
- The Monte Carlo simulation process should be detailed, as besides the achieved results, there is nearly no information on how the process has been performed.
*Thank you for the suggestion. We add the following description:*
* “ We thus proposed a different approach that takes into account the demand fluctuations, and which involves a Monte Carlo simulation. In this approach, we estimated the optimal PV size using NPV criteria (eqs. (3)-(7)) on a single synthetic profile. This procedure was repeated N times. The result for the optimal PV size is the average of the N outputs. Figure 23 shows the flowchart of this algorithm used for the MC simulation. Optimal PV sizes were then compared for different number of runs N, where N ranged from 10 up to 1500. *
*Figure 23 is linked here.*

 
*Figure 23. Flowchart of the MC simulation to determine the PV optimal size.* *When defining the  profiles  we assumed an influx of one user/day at slow CPs and nine users/day at fast CPs in a typical CI (two slow CPs and three fast ones) to create synthetic profiles as those in* *Figure 21* *(top panel). To simulate the uncertainties on the arrivals, we added white noise to the NPDs of* *Figure 19*”

  

- The overall organization of the document does not help the reader, in specific, the places where figures and tables are presented are often highly disconnected with the pieces of text that discuss them. This makes the analysis of such a large number of analyses even more difficult.

*Thanks a lot for bringing up this point. In the draft version, we provided the editor, the order of the figure as well as the one of the tables was consistent with the different paragraphs/sessions of the text. However, it is possible the final layout chosen by the journal, for some editing reasons might have moved some figures inappropriately within the main text. We are going to make a remark to the editor to see if this issue can be overcome and therefore the readability of the text can be improved. In addition, from our side, we have reviewed the entire structure of the text to assure that all figures and table are consistent with the text.*

- The results on the potential advantages and sizing of the PV system should be more clearly discussed.
*Thank you for your comment. We add the following sentence:*
  “ *The presence of a PV system reduce the dependence on the grid and can contribute to lower* *pollutant emissions* *, especially when coupled with a storage system* *31* ^,^^32^.”
   
*And references 31 and 32:*

- *Krim Y, Sechilariu M, Locment F. PV Benefits Assessment for PV-Powered Charging Stations for Electric Vehicles.* Applied Sciences *. 2021; 11(9):4127. https://doi.org/10.3390/app11094127*

- *Yang M, Zhang L, Zhao Z, Wang L: Comprehensive benefits analysis of electric vehicle charging station integrated photovoltaic and energy storage.* *Journal of Cleaner Production. 2021; 302:126967. 10.1016/j.jclepro.2021.126967.*

# Peer review: Reviewer response for version 1

**DOI:** 10.21956/openreseurope.15491.r28229

**Published:** 2022-01-10

**Authors:** Sandford Bessler

**Affiliations:** 1 TU Wien, Wien, Austria

The authors address in this paper the planning of public EV charging stations. Instead of using mobility data, they use collected data at existing charging stations in Barcelona and Turku and generate the needed daily/yearly demand distribution.

 Some remarks to the readability:

- In the introduction, it is a good practice to mention clearly the goals of the research and the contributions beyond the state of the art.
- Start the “Results and discussion” Chapter by presenting shortly the processing workflow as in Fig 20.
- A central result in section “Simulation results” is to build from the data set a kernel density estimation (KDE). The method has to be better described and/or references have to be added.

The optimization problem presented after Figure 21 “To check the impact of the profiles used as input in sizing problems” comes unexpected in the middle of the section. It solves the economic investment in a PV installation at a charging station.

 The authors should mention the motivation to choose this problem, given that the charging infrastructure is only lightly loaded (see last remark).

 In general adding PV without storage is not efficient, because the charging demand is in general not flexible in public CS, and the PV generation peak is not the same as the demand peak, as shown in Fig 22.

 However, as an application for using the (stochastic) charging load profiles, the optimization for sizing the PV installation for a CI is well formulated.

- Provide at the beginning the reason for using the Monte Carlo instead the averaged profiles: “when the usage rate of the charging infrastructure is low, data fluctuation must be considered, as it might heavily affect the load profiles.”

- Please specify: When applying Monte Carlo simulation, how is the stochastic profile defined, is the optimal PV size estimated still by solving (2-6)?

Here I have to mention a weakness of the paper in addressing or at least mentioning the real problems of charging infrastructure planning, which could make use of the computed synthetic load profiles  (Fig.21).

 Due to low EV penetration, the datasets didn’t show any overload of the CSs in the two cities, but resource problems would arise with the demand increase. Therefore, some questions for further research are:

- How much can we upscale the future charging load (higher EV penetration) without changing the grid connection capacity (kW) of the CI?
- How much can we further increase the charging load if technologies such as smart charging with variable charging rates etc. are available at the CI and EV?

Is the study design appropriate and does the work have academic merit?

Yes

Is the work clearly and accurately presented and does it cite the current literature?

Partly

If applicable, is the statistical analysis and its interpretation appropriate?

I cannot comment. A qualified statistician is required.

Are all the source data underlying the results available to ensure full reproducibility?

Yes

Are the conclusions drawn adequately supported by the results?

Yes

Are sufficient details of methods and analysis provided to allow replication by others?

Partly

Reviewer Expertise:

EV charging optimization, smart grids, renewable energy communities

I confirm that I have read this submission and believe that I have an appropriate level of expertise to confirm that it is of an acceptable scientific standard, however I have significant reservations, as outlined above.

# Response

**Published:** 2022-02-04

**Authors:** Natascia Andrenacci

**Affiliations:** Ente per le Nuove Tecnologie, l'Energia e l'Ambiente (ENEA), Italy

*The authors would like to thank the reviewer for his comments and valuable input.* *In the following, we have tried to respond to the observations point by point. Our responses are shown in italics, and changes made to the text of the article are underlined.* :

 In the introduction, it is a good practice to mention clearly the goals of the research and the contributions beyond the state of the art.

*Thank you for this suggestion. We add the following sentence to the introduction:*

*“This work takes into consideration the data collected in some public charging points in the cities of Barcelona and Turku. The results of the statistical analysis of the data allow synthesizing profiles that represent an input to determine the optimal size of a renewable source (photovoltaic) to be used in a charging station. This work aims to underline the impact that profiles obtained with different methods can have on determining the optimal investment for a renewable source, especially in an early phase of the exploitation of the charging infrastructure.”*

 Start the “Results and discussion” Chapter by presenting shortly the processing workflow as in Fig 20.

*Thank you for this valuable suggestion. Indeed, it is more appropriate to introduce the workflow in an earlier stage. We moved Figure 20 from its original position to the end of the section “Methods”, along with a brief description of the procedure, which we report below for convenience:*

*“We performed the analysis of the main statistical parameters relating to the data sets, such as the average and standard deviation of the duration of the charge and the energy exchanged, as a function of external variables such as the start time of the charge, the days of the week, and seasonality. Furthermore, we investigated the correlations between EV models and data set parameters, as well as the correlation between parameters. The statistical analysis outputs allow generating different possible charging demand scenarios, which can be the input of the charging infrastructure design and demand management algorithms. In particular, the procedure used builds the hourly distribution of arrivals using the non-parametric fitting method. Moreover, we assumed that the duration of the charge and energy demand in a given time interval both follow a Gaussian distribution, with mean and variance values derived from the data sets for the selected time interval. We modelled the stochastic nature of the arrivals adding a white noise signal to the NPD. A similar variability has been added to the charge duration and energy distributions. This approach allows generating load demand scenarios for different levels of EV penetration, by varying the average number of users per day. The workflow of the generation process is illustrated in Figure 1.*

*The synthetic profiles represent the instantaneous average power demand at the CS. The average power is defined as the ratio between the energy and charge duration values for the given time interval. We assumed that the charging time reported in the data sets corresponds to the time it takes to deliver the charging energy to the vehicle.”*

*At the beginning of the “Results and discussion” Chapter we added the following: “The results of the statistical analysis of the Barcelona and Turku datasets are presented below. We illustrate the criteria for excluding some records from subsequent analysis. We also report the results for the CSs' occupation, the correlations between energy and duration of the charges, and the distribution of the average power at the CPs. For the Barcelona dataset, the possible correlation between the EV battery size and the charged energy is also investigated.*

*A procedure for creating charging profiles from statistical analysis is presented. These profiles are used as input in a size optimization algorithm of a photovoltaic system, and the result is compared with that obtained with other synthesis profiles. Based on the feedback with the data, we proposed a size optimization procedure that provides better outcomes for low CS utilization scenarios.* *”*

 A central result in section “Simulation results” is to build from the data set a kernel density estimation (KDE). The method has to be better described and/or references have to be added.

*Thank you for pointing out this missing definition. We add the following description to the text:*

*Among the nonparametric estimates, we chose a kernel density estimation (KDE), which estimates the probability density function (PDF) of a random variable making use of a kernel function K(x,h), and a smoothing parameter, h, called the bandwidth. It allows creating a smooth curve from a dataset, from which inferences about the population can be made. The kernel function is a generic function with the following properties:*

- *Symmetric with respect to zero: K(x,h)=K(-x,h);*
- *Normalized: ∫_(-∞)^∞▒* *〖* *K(x,h)dx=1* *〗* *;*
- *lim┬(x→∞)⁡* *〖* *K(x,h)=lim┬(x→-∞) K(x,h)=0* *〗* *.*

*The smoothness of the resulting curve depends on the bandwidth parameter. A large bandwidth leads to a very smooth (i.e. high-bias) density distribution, while a small bandwidth leads to an unsmooth (i.e. high-variance) density distribution. KDE  is made by weighting the distances of all data from each value of the independent variable. Mathematically, the PDF estimate at a point x within a group of points {x_1,x_2,…,x_N} is given by:* *f* *h* *x*= *i*=1 *N* *K*( *x*- *x* *i*, *h*)

*[Illustration of equation can be seen here]*

 The optimization problem presented after Figure 21 “To check the impact of the profiles used as input in sizing problems” comes unexpected in the middle of the section. It solves the economic investment in a PV installation at a charging station.

*Thank you for your remark. We reformulate the sentence to make it clearer: “Average data shown in Figure 21 smoothed the peaks and distributed the charging requests more evenly. This could affect the outcome of an optimal size procedure for a CI, especially if auxiliary services were present, such as a renewable energy source and storage systems. In the present work, to check the impact of the profiles used as input in sizing problems, we considered the CI of type b) for AMB and we evaluated the optimal size of a photovoltaic (PV) system to install in the CI according to different load profiles. We have considered a scenario that sees a photovoltaic (PV) system without storage to power an IC, as it appeared to be the solution used in the city for public charging.”*

 The authors should mention the motivation to choose this problem, given that the charging infrastructure is only lightly loaded (see last remark).

*The reasons for this choice reside in the fact that one charging infrastructure included in the study had photovoltaic panels without storage. Moreover, the presence of a PV system without storage emphasizes the effects of using more or less smoothed artificial profiles, which was our goal in this paper.*

 In general adding PV without storage is not efficient, because the charging demand is in general not flexible in public CS, and the PV generation peak is not the same as the demand peak, as shown in Fig 22.

*We do agree that storage is necessary to exploit the PV energy more efficiently. However, in the public charging stations we analyzed, battery storage is usually not present. In another work to be published, we apply the optimization procedure to a complete PV+BES system. To address this point we add the following sentence after fig.22: *“ *We stress out that the analysis was made for a specific case reflecting the observed situation in AMB. The presence of a storage system modifies the results and the convenience of the investments.*”

 However, as an application for using the (stochastic) charging load profiles, the optimization for sizing the PV installation for a CI is well formulated.

 Provide at the beginning the reason for using the Monte Carlo instead the averaged profiles: “when the usage rate of the charging infrastructure is low, data fluctuation must be considered, as it might heavily affect the load profiles.”

*Thank you for the suggestion. We reformulated the sentence that introduces the method as the follows: “We thus proposed a different approach that takes into account the demand fluctuations, and which involves a Monte Carlo simulation. In this approach, we estimated the optimal PV size using NPV criteria (eqs. (3)-(7)) on a single synthetic profile. This procedure was repeated N times. The result for the optimal PV size is the average of the N outputs. Figure 23 shows the flowchart of this algorithm used for the MC simulation.. Optimal PV sizes were then compared for different number of runs N, where N ranged from 10 up to 1500.” (please note that equations have been renumerated).*

 Please specify: When applying Monte Carlo simulation, how is the stochastic profile defined, is the optimal PV size estimated still by solving (2-6)?

*This point has been addressed in the sentence posted above.*

 Here I have to mention a weakness of the paper in addressing or at least mentioning the real problems of charging infrastructure planning, which could make use of the computed synthetic load profiles  (Fig.21).

*Thank you for your annotation. Indeed, the problem of optimal sizing of charging stations is central. However, in our work, we wanted to emphasize the differences among the results obtained using different demand evaluation approaches. Thus, we preferred to compare the results for the optimal PV size for different profiles in the existing CI configuration and evaluate them against the actual demand at the stations. We added the following sentence: “In general, the optimization problem involves the charging structure. However, we limited our analysis to the size of the renewable source, to be able to evaluate the results of the optimization* *for different synthetic profiles on the real data, using the load curve of an existing station”.*

 Due to low EV penetration, the datasets didn’t show any overload of the CSs in the two cities, but resource problems would arise with the demand increase. Therefore, some questions for further research are:

 ​​​​

 How much can we upscale the future charging load (higher EV penetration) without changing the grid connection capacity (kW) of the CI?

 How much can we further increase the charging load if technologies such as smart charging with variable charging rates etc. are available at the CI and EV?

*Thank you so much for these interesting insights for future research. The answer to both cannot be exhausted in a few sentences, and critically depends on the development of the charging network. The issue of smart charging is very interesting and will be addressed in a future paper. We add a sentence in the Conclusions “ The management of EV demand addresses several issues, especially for the load increases due to a broader diffusion of electric mobility. Smart charge strategies, such as variable charging rates, can help mitigate the impact on the electric grid. This topic will be addressed in future work, using ancillary data and analysis from the present case study.”*
